# Vagal innervation limits brain injury by inhibiting gut-selective integrin-mediated intestinal immunocyte migration in intracerebral hemorrhage

**DOI:** 10.7150/thno.101680

**Published:** 2024-10-28

**Authors:** Peiji Fu, Yan Zong, Yinming Dai, Li Zhu, Shuai Chen, Yousef Rastegar-Kashkooli, Junmin Wang, Jiewen Zhang, Jian Wang, Chao Jiang

**Affiliations:** 1 Department of Neurology, The People's Hospital of Zhengzhou University & Henan Provincial People's Hospital, 450003, Zhengzhou, P. R. China.; 2 Department of Neurology, The Fifth Affiliated Hospital of Zhengzhou University, 450052, Zhengzhou, P. R. China; 3 The Laboratory of Cerebrovascular Diseases and Neuroimmunology, The Fifth Affiliated Hospital of Zhengzhou University, 450052, Zhengzhou, P. R. China.; 4 Department of Human Anatomy, School of Basic Medical Sciences, Zhengzhou University, 450001, Zhengzhou, P. R. China.

**Keywords:** Intracerebral hemorrhage, Vagus nerve, Intestinal immunocyte trafficking, α7nAChRs, Gut-selective integrin, Immunomodulatory therapy

## Abstract

**Rationale**: The vagus nerve, which connects the brain and gastrointestinal tract, helps to maintain immune balance in the intestines. Gut-specific integrins, on the other hand, help to keep immune cells in the intestines. Since immune cells from outside the intestines can significantly affect the outcome of strokes, we investigated how immune cells from the intestines affect the immune response in the brain during intracerebral hemorrhage (ICH).

**Methods**: We aimed to examine the impact of vagal innervation on intestinal immunocyte trafficking and its influence on ICH outcomes using Kikume Green-Red (KikGR) and wildtype (WT) mice, with or without prior subdiaphragmatic vagotomy (SDV). Furthermore, we sought to elucidate the regulatory effects of vagal innervation on intestinal immunocyte trafficking by activating α7 nicotinic acetylcholine receptors (α7nAChR) in WT mice that underwent ICH after SDV. Additionally, we explored the potential intermediary role of gut-selective integrins in cholinergic transmitters-mediated intestinal immunocyte trafficking. Our methodology encompassed *in vivo* fluorescence imaging, flow cytometry, Western blotting, immunofluorescence staining, histopathology, and behavioral assessments to evaluate the outcomes.

**Results**: Our findings reveal that during the acute phase of ICH, intestinal immunocytes migrated to various anatomical locations, including the circulation, hemorrhagic brain, meninges, and deep cervical lymph nodes. Pertinently, SDV resulted in diminished expression of α4β7 and αEβ7 integrins on immunocytes, leading to heightened intestinal immunocyte trafficking and exacerbated ICH outcomes. Conversely, the administration of α7nAChR agonists countered the adverse effects of vagotomy on α4β7 and αEβ7 integrin expression, thereby constraining the migration of immune cells from the intestines after ICH. The implication of α4β7 and αEβ7 integrins in this setting was supported by the ineffective influence of α7nAChR agonists on the trafficking of intestinal immunocytes enhanced by administering beta-7 integrin antagonists, such as etrolizumab. It was further supported by the exacerbated ICH outcomes by administering beta-7 integrin antagonists like etrolizumab alone.

**Conclusion**: The identification of vagus nerve-mediated modulation of α4β7 and αEβ7 integrin expression in the trafficking of immune cells within the intestinal tract holds significant implications. This discovery presents new opportunities for developing therapeutic interventions for ICH and stimulates further investigation in this area.

## Introduction

Intracerebral hemorrhage (ICH) is a prevalent subtype of stroke, accounting for 20%-30% of all strokes 1, 2. Unfortunately, unlike ischemic stroke, no significant developments have been made in treating ICH 1, 2. Further research is needed to understand the brain injury and repair mechanisms and develop new treatment options for ICH. One promising strategy is to regulate inflammation, as secondary damage caused by the inflammatory response can worsen neurologic deficits after ICH. Therefore, exploring neuroinflammatory regulation as a potential treatment for ICH is essential 3, 4.

The innate glia get activated in response to inflammation caused by ICH and immunocytes infiltrate the damaged brain tissue 5, 6. These immunocytes include neutrophils, monocyte-derived macrophages, lymphocytes, and others 7, 8. T lymphocytes have multiple phenotypes and play a critical role in the adaptive immune response 2, 9. Regulatory T cells show a neuroprotective effect, but other subpopulations, such as T-helper 1 cells and cytotoxic T cells, may worsen during the acute phase of ICH 7, 8. Therefore, preventing toxic lymphocyte migration from the bloodstream or other immune organs to the brain is a promising way of treating ICH 10, 11. However, further investigation is needed to determine these cells' exact origin and mechanism of migration.

The gastrointestinal tract, a major reservoir of immune cells, plays a crucial role in regulating immune response beyond the organ itself, especially during stressful situations 8. Lymphocytes are believed to travel from the digestive system to the brain 12-14, which may be triggered by gut microbiota dysbiosis 12, 13. The communication network between the gut and brain is intricate, and the vagus nerve plays a role in its regulation 15, 16. It is unclear whether the vagus nerve also governs the movement of immunocytes from the intestines to the brain, which can cause neuroinflammation. More research is required to explore this possibility, particularly in stroke cases.

Additional evidence has also shown that stimulating the vagus nerve helps alleviate neuroinflammation after stroke 8. This effect is due to the activation of α7 nicotinic acetylcholine receptors (α7nAChR) in immune cells, including lymphocytes 8. To investigate the effects of the vagus nerve on the trafficking of immunocytes from the mesenteric lymph nodes (MLNs) to the brain of mice with ICH subjected to vagotomy, we used transgenic mice in which cells are labeled with the photoconvertible protein (Kikume Green-Red, KikGR) 17, 18. As α4β7 integrin facilitates the homing of leukocytes to the gut and αEβ7 integrin retains lymphocytes in mucosal tissues of the gut 19, we further investigated the influence of vagotomy, α7nAChR agonists, and beta-7 integrin antagonists on intestinal immunocyte trafficking in wildtype mice with ICH.

Our investigation revealed that vagal innervation, which regulates intestinal-selective leukocyte homing and retention by preserving α4β7 and αEβ7 integrin expression via the acetylcholine pathway, can impede intestinal lymphocyte trafficking and mitigate brain injury and functional impairments in mice with ICH. These findings hold great promise for the development of novel immunoregulatory interventions for the treatment of ICH.

## Materials and Methods

### Animals, experimental groups, and treatment regimens

The Animal Ethics and Use Committee of Zhengzhou University approved all procedures (ZZU-LAC20210924), and all animal experiments were conducted according to the ARRIVE guidelines. We used C57BL/6 male wildtype (WT) mice (8-10 weeks, 23-27 g) obtained from the Laboratory Animal Center of Zhengzhou University and male Kikume Green-Red (KikGR) transgenic mice (8-10 weeks, 22-26 g) bred by us but generated by Cyagen Bioscience Inc. (Suzhou, China). The animal rooms were pathogen-free and kept at a stable 21-25 °C temperature, humidity of 58%-68%, and a light/dark cycle ratio of 12 : 12 h.

To assess how the vagus nerve affects immunocyte trafficking from the intestines to the brain, we used KikGR and WT mice that underwent ICH 20 d after vagotomy 20, 21. KikGR mice were randomly divided into two groups using computer-generated random numbers: (1) Sham vagotomy + ICH group, where mice received sham vagotomy and ICH, and (2) Vagotomy + ICH group, where mice underwent vagotomy and ICH. To evaluate whether vagotomy aggravates brain injury by enhancing neuroinflammation, we randomly assigned WT mice to 1 of 4 groups: Sham vagotomy + Sham ICH group, where mice received sham vagotomy and sham ICH; Vagotomy + Sham ICH group, where mice underwent vagotomy and received sham ICH; Sham vagotomy + ICH group, where mice received sham vagotomy and ICH; and Vagotomy + ICH group, where mice underwent vagotomy and ICH.

To investigate whether the cholinergic pathway regulates immunocyte trafficking through the vagus nerve, we randomly assigned WT mice to 1 of 3 groups: (1) Sham ICH + vehicle, (2) Vagotomy + ICH + vehicle, and (3) Vagotomy + ICH + α7nAChR agonist. Furthermore, our objective was to confirm vagal innervation inhibits intestinal immunocyte trafficking and improves ICH outcomes by reserving α4β7 and αEβ7 integrin expression. To do so, we randomly assigned WT mice to 1 of 3 groups: (1) ICH + vehicle, (2) ICH + β7 integrin antagonist, and (3) ICH + β7 integrin antagonist + α7nAChR agonist. Concerning studies associated with SDV, ICH was induced 20 days after sham or vagotomy, and the α7nAChR agonists (PHA-543613, PZ0135, Sigma-Aldrich) were administered intraperitoneally 1 h after ICH at 12 mg/kg dissolved in saline with 5% DMSO 20-22. As for studies about the function of α4β7 and αEβ7 integrins, the β7 integrin antagonists (Etrolizumab, HY-P9984, MedChemExpress) and α7nAChR agonists (PHA-543613, PZ0135, Sigma-Aldrich) were administered intraperitoneally 1 h after ICH at 5 mg/kg dissolved in saline and 12 mg/kg dissolved in saline with 5% DMSO, respectively 19, 22. The vehicle group received identical volumes of saline or saline containing 5% DMSO.

### Subdiaphragmatic vagotomy (SDV) mouse model

We used the SDV mouse model for the study 23, 24. Mice were anesthetized with 3.0% isoflurane and kept at 1% through a nasal cone in a mixture of 80% nitrogen and 20% oxygen. A bilateral SDV or sham operation was conducted in the mice. A 3 cm middle longitudinal abdominal incision was made below the xiphisternum during the procedure. The costal arc was then retracted using a vascular clamp, and the liver and stomach were gently pushed aside with small cotton balls dampened with sterile saline to expose the esophagus. The vagus nerve's dorsal and ventral truncal branches along the subdiaphragmatic esophagus were separated and transected under a surgical microscope (SMZ800N, Nikon, Japan). Mice in the sham vagotomy group underwent the same surgery, but the subdiaphragmatic vagus nerves were not transected (Figure S1A). Throughout surgery, the rectal temperature of the mice was maintained at 37.0 ± 0.5 °C. After surgery, the mice were allowed to recover for 20 days with free access to food and water. We measured food intake to confirm the success of SDV 25, since the cholecystokinin octapeptide (CCK-8) inhibits food intake by stimulating vagal afferent fibers. Eight days after SDV, mice received CCK-8 (40 μg/kg, HY-P0093A, MedChemExpress) dissolved in saline by tail vein injection 21. The mice were then kept individually in cages where the weight of the food was weighed in advance, and the net amount of food consumed by the mice was calculated for 24 h. The absence of CCK-8-induced feeding suppression determined the success of SDV (more details are available in Figure S1B).

### Photoconversion of KikGR mice

The Kikume-Green photoconvertible fluorescent protein in KikGR mice can be converted to Kikume-Red after exposure to near-UV light (350-420 nm). In this study, homozygous KikGR mice were anesthetized with 3.0% isoflurane inhalation, and MLN photoconversion was performed as previously described 12, 14. We covered the entire body with sterile aluminum foil, sterilized the abdomen, and made a 1-cm abdominal incision through an opening in the aluminum foil to expose the mesenteric lymph node (MLN). We covered the abdominal cavity with sterile aluminum foil, except for the isolated MLN, to prevent neighboring tissues from being irradiated. The MLNs were then illuminated with a defocused violet laser light source (405 nm, peak power 4.5 mW, 14 × 37 mm beam range, Hongdu Electronics, China) for 15 min at 5 cm (Figures S1C-D) while care was taken to keep the intestinal tissue hydrated with sterile saline throughout the procedure. Seven days after photoconversion, MLN immunocytes were extracted and prepared for flow cytometric analysis (*n* = 5 mice per group). We analyzed the percentage of photoconverted cells (KikR^+^ in red) in the populations of CD45^+^ cells. Furthermore, we monitored intestinal monocyte trafficking 3 d after ICH in KikGR mice undergoing photoconversion 4 d before.

### ICH Mouse Model

The ICH model involves injecting collagenase into the left striatum of mice to induce bleeding 9, 26. We secured the mice to a stereotactic head frame (RWD Life Science, China) for surgery and drilled a small burr hole. Then, using a 1 μL Hamilton microinjection needle, collagenase VII-S (0.075 U in 0.5 μL of saline, C2399, Sigma-Aldrich) was injected at specific coordinates (0.8 mm anterior and 2.1 mm lateral of the bregma, and 3.1 mm deep). After the collagenase was administered for more than 5 min, the needle was held for 10 min to prevent backflow and then slowly withdrawn. Sham mice underwent the same procedure but do not receive collagenase injections. After injection, the burr hole is sealed with Super Glue (Loctite, Germany). The mice are maintained at a constant rectal temperature of 37.0 ± 0.5 °C throughout the experimental and recovery periods. Mice that died before the end of the study are excluded, while the remaining animals are included in the final analysis.

### Brain injury volume, swelling, residual lesion, and atrophy

Following established protocols 9, 27, our experimental procedure involved anesthetizing the mice and transcardially perfusing them with saline and 4% paraformaldehyde (PFA) on day 3 or 28 after ICH. Brains were removed and stored at 4 °C in 4% PFA overnight, followed by dehydration with 20% sucrose and 30% sucrose solutions and embedding in optimal cutting temperature compounds (Tissue-Tek OCT Compound, 4853, SAKURA). Using a cryostat (Leica, Germany), we cut the samples into a 50 μm section and 12 30 μm sections, covering ten cycles from the olfactory bulbs to the visual cortex. The 50 μm coronal sections, spaced 360 μm apart, were stained with Luxol fast blue (LFB, Solvent Blue 38, S3382, Sigma-Aldrich) for myelin and Cresyl Violet (CV, Cresyl Violet acetate, C5042-10G, Sigma-Aldrich) for neurons. This allowed us to measure brain lesion volume, white matter injury, brain swelling, and atrophy. The 30-μm sections were stored in cryopreservation solution at -20 °C for immunofluorescence and LFB staining.

Brain lesion volume was quantified with SigmaScan Pro software (version 5.0.0 for Windows; Systat, San Jose, CA, USA) on day 3 or 28 after ICH (*n* = 10 mice per group). We identified the injured extent of the brain lesion by locating areas lacking LFB/CV staining in the 50-μm sections. We calculated the lesion volume in cubic millimeters by multiplying the damaged area by the distance between the slices 9, 27.

Brain swelling was measured by determining hemisphere enlargement on day 3 after ICH (*n* = 10 mice per group). Using SigmaScan Pro software, we quantified volumes of the ipsilateral and contralateral hemispheres. Brain swelling (%) was expressed as [(ipsilateral hemisphere volume — contralateral hemisphere volume)/contralateral hemisphere volume] × 100% 9, 27.

We subtracted the ipsilateral hemisphere volume to calculate brain atrophy induced by grey matter and white matter injuries in the hemorrhagic hemisphere on day 28 after ICH (*n* = 10 mice per group). We expressed the result as a percentage of the contralateral hemisphere volume: [(contralateral hemisphere volume - ipsilateral hemisphere volume) / contralateral hemisphere volume] × 100% 9, 27.

### Brain water content

We investigated the evolution of brain edema 9. Three days after ICH, the mice were anesthetized and decapitated (*n* = 6 mice per group). The whole brain was removed and divided into three parts: the ipsilateral striatum, the contralateral striatum, and the cerebellum (as an internal control). The weight of the samples was measured immediately to obtain the wet weight and then kept in an electric blast drying oven at 100 °C for 24 h to get the dry weight. Finally, the brain water content was calculated using the formula [(wet weight - dry weight) / wet weight] × 100% 9, 28.

### Assessment of neurologic deficits

We used the modified neurologic deficit score (NDS) and the corner turn test (CTT) to evaluate the long-term neurologic function of mice following ICH. Our assessments were conducted on days 1, 3, 7, 14, and 28 after ICH (*n* = 10 per group). The NDS consists of six elements: body symmetry, gait, circling behavior, front limb symmetry, climbing, and compulsory circling. Each component is scored on a scale of 0-4, resulting in a total range of 0-24 9, 27. To conduct the CTT, we tracked the number of times the mice turned left in a 30-degree angled device. We replicated this test 10 times per time point and expressed the results as a percentage 7, 9.

### White matter damage and myelin loss

To evaluate white matter damage, we collected 30-µm brain sections on day 28 after ICH (*n* = 10 mice per group). From each preserved brain, we selected 3 sections at similar locations that were then stained with LFB and examined for intact myelin in the perihematomal region 9, 29. Using light microscopy, we photographed 4 comparable fields per section with the same exposure level. Subsequently, we used ImageJ software (ImageJ 1.4, NIH, USA) to quantify the areas covered by LFB staining of 12 locations per mouse (4 fields × 3 sections). We then averaged these values and divided them by the total area of white matter examined 9, 29.

### Immunofluorescence

To track the migration of immunocytes in the intestine in the context of a hemorrhagic brain, KikGR mice were subjected to sham surgery or MLN photoconversion 4 days before ICH. Three days after ICH, the mice were anesthetized and perfused with saline and 4% paraformaldehyde (PFA) through the heart (*n* = 3 mice per group). The entire brain was removed, embedded in paraffin, and sectioned into 10-μm coronal slices 30. These brain slices, taken at similar locations, were scanned using the Pannoramic Digital Slide Scanner (Pannoramic MIDI II, 3DHISTECH, Hungary) to photograph photoconverted KikR^+^ cells in the perihematomal regions and qualitatively observe the movement of intestinal immunocytes.

After ICH, the activation of microglia and astrocytes and the infiltration of neutrophils, the most abundant immunocytes in circulation, become evident by day 3 9. The cellular inflammatory response within the hemorrhagic brain was evaluated by performing immunofluorescent staining to quantify microglia, astrocytes, and neutrophils (*n* = 6 mice per group) 9. For this purpose, 3 coronal 30-μm brain sections were selected from each mouse at the same levels from the cryopreservation solution and washed in PBS for 50 min for immunofluorescence staining. The selected sections were blocked in 3% bovine serum albumin (BSA) for 60 min at room temperature and then incubated with primary antibodies overnight at 4 °C. The primary antibodies used were rabbit anti-glial fibrillary acid protein (GFAP, astrocyte marker, 1:200; 16825-1-AP, Proteintech), rabbit anti-ionic calcium-binding adapter molecule 1 (Iba-1, microglia/macrophages, 1:1000; 019-19741, Wako), and rabbit anti-myeloperoxidase (MPO, neutrophil manufacturer, 1:150; ab9535, Abcam).

After being washed 3 times with PBST for 5 min each, the sections were incubated for 1 h at room temperature with the following fluorochrome-conjugated secondary antibodies: goat anti-rabbit 488 (1:1000; A-11034, Invitrogen); goat anti-rabbit 594 (1:10; R37117, Invitrogen). The sections were rewashed with PBS for 15 min at room temperature. Brain sections omitted from the primary antibody incubation step were identically processed for negative controls. We observed the perihematomal area of each mouse under a fluorescence microscope (Ni-U, Nikon, Japan) and analyzed 12 locations (4 fields of view × 3 sections). Reactive microglia/macrophages were defined as the cells that were spherical, amoeboid, or rod-shaped, had a diameter of > 7.5 μm in at least one direction, had short and thick processes, and exhibited strong Iba1 immunofluorescence. On the other hand, resting microglia/macrophages were characterized by small cell bodies (< 7.5 μm in diameter) with long processes and weak Iba1 immunofluorescence 9. We defined reactive astrocytes as intense GFAP immunofluorescence with longer and thicker processes in the injured hemispheres 9. The number of positive cells was analyzed by ImageJ (ImageJ 1.4, NIH, USA) and averaged as the number of positive cells per square millimeter.

### Fluoro-Jade B staining

On day 3 after ICH, we used fluorescent Jade B (FJB, TR-150-FJB, Biosensis) staining 9, 29 to identify degenerating neurons on 3 30-μm sections of each mouse (*n* = 6 mice per group). Subsequently, stained sections (4 fields × 3 sections per mouse) were observed and photographed under a fluorescence microscope (Ni-U, Nikon, Japan). To quantify FJB-positive cells, we counted the number per square millimeter, like the measurement of immunoreactive cells. The results were presented as the number of FJB-positive cells per square millimeter.

### Western blotting

Mice were anesthetized with isoflurane and transcardially perfused with saline 24 h after ICH (*n* = 5 mice per group). The brains were removed and placed in chilled saline 9, 29. Brain tissue around the hematoma was homogenized with radioimmunoprecipitation assay (RIPA) lysis buffer and a PMSF protease inhibitor (RIPA/PMSF, 100:1, Solarbo). After centrifugation at 14,000 g for 30 min at 4 °C, total protein was quantified in each sample using the enhanced BCA protein assay kit (P0010S; Beyotime). Protein samples were heated at 99 °C for 10 min. Then, equal amounts of protein were separated with 12% sodium dodecyl sulfate (SDS)-polyacrylamide gel electrophoresis (PAGE) and transferred to polyvinylidene difluoride (PVDF) membranes. The membranes were sealed with 5% skim milk for 2 h at room temperature and then incubated with primary antibodies overnight at 4 °C: rabbit anti-high mobility group box 1 (HMGB1, 1:800; ab18256, Abcam), rabbit anti-IL-1β (IL-1β, 1:600; ab200478, Abcam), rabbit anti-IL-6 (IL-6, 1:1000; 21865-1-AP, Protein), rabbit anti-TNF-α (TNF-α, 1:1000; 17590-1-AP, Protein), mouse anti-β-actin (β-actin, 1:6000; 66009-1-Ig, Proteintech). The membranes were then washed 3 times in TBST for 10 min each and incubated with secondary antibodies: goat anti-rabbit (HRP-conjugated Affinipure goat anti-rabbit IgG(H+L), 1:10000; SA00001-2, Proteintech) and goat anti-mouse (HRP-conjugated Affinipure goat anti-mouse IgG (H+L), 1:6000; SA00001-1, Proteintech) for 1 h at 37 °C. The protein signal was visualized with the ECL chemiluminescence reagent kit (KF001; Affinity) and photographed with the Bio-Rad imaging system. Finally, the results were expressed as the target grey value over the internal reference grey value analyzed with ImageJ software, and the internal reference was β-actin.

### *In vivo* fluorescence imaging

An *in vivo* optical imaging system is utilized to visualize fluorescent or bioluminescent signals in live animals without invasive procedures 31, 32. The photomodulatable fluorescent protein KikGR, expressed widely in transgenic mouse cells, allows real-time monitoring of cell migration. After being irradiated with UV at a specific site, KikG (green) can be converted to KikR (red) 33. We used KikGR mice that underwent sham vagotomy or SDV (*n* = 5 per group) to detect green and red fluorescence distribution and intensity in the heads and bodies immediately before and 4 (immediately before ICH) and 7 days (3 days after ICH) after photoconversion in the MLNs (Perkin Elmer, USA). On day 3 after ICH, KikGR mice were anesthetized with isoflurane and transcardially transcradially perfused with saline. The brain, deep cervical lymph nodes (dCLNs), and MLNs were removed to detect the distribution and intensity of green and red fluorescence in these areas. We used the IVIS® Spectrum system and Living Image 4.4 software (PerkinElmer Health Sciences, USA) to quantify fluorescence intensity and assess tissue distribution of KikR cells from the MLNs.

### Cell isolation from brain tissue and meninges

Following anesthesia, sacrifice, and transcardial perfusion, we collected the heads of mice. The brain tissue and meninges were extracted and stored in 1 x PBS on ice (*n* = 5-6 mice per group). Using an established protocol, we prepared single-brain cell suspensions using the Adult Mouse Brain Tissue Dissociation Kit (DHABE-5003, RWD Life Sciences, China) 9, 26, 34. The brain tissues from each hemorrhagic hemisphere were cut into small pieces, and enzymatic dissociation was carried out using enzymes A and B from the enzyme digestion kit in a tissue processing tube (SCT-100, RWD Life Sciences, China). Then, mechanical, enzymatic dissociation of the tissue was performed using the M_ABrain_Heater_1 model of the Single Cell Suspension Preparator (DSC-400, RWD Life Sciences, China). The homogenate was filtered through a 70 μm cell strainer (BS-70-CS, Biosharp) to remove the debris and erythrocytes, and the reaction was stopped by adding 1 × PBS. Cells were washed, disaggregated, and centrifuged at 400 g for 10 min at 4 °C before resuspending in 1 × PBS for flow cytometric analysis. Additionally, the meninges were mechanically homogenized in a buffer containing 0.41 U/ml LeberaseTM (5401119001, Roche) and 60 U/ml DNAseI (10104159001, Roche) for 1 h 35. Meninge homogenate was filtered through a 70 μm strainer, and the reaction was stopped by adding 5 ml 1 × PBS. Cells were resuspended in 1 × PBS for flow cytometric analysis after gradients were centrifuged at 280 g for 10 min.

### Cell isolation from blood and lymphoid organs

To minimize animal use, animals were used to isolate cells from brain tissue, and meninges were also used to isolate cells from peripheral blood, MLNs, and Peyer's patches (*n* = 6 mice per group). Before sacrificing the mice, we collected peripheral blood from the femoral vein and placed it in tubes containing sodium heparin. To prepare cells for analysis, we added erythrocyte lysis buffer (R1010, Solarbio) and incubated them for 5 min at room temperature. Subsequently, we added 10 ml 1 × PBS to the tubes 12 and centrifuged the cell suspensions at 500 g for 10 min before analyzing them with flow cytometry.

For KikGR mice, we focused on dCLNs and MLNs, while for WT mice, we isolated MLNs and Peyer's patches after transcardial perfusion. Using a premoistened 70-µm cell strainer, we gently homogenized the lymphoid tissues with a well-trimmed 1ml syringe plunger. We then washed the strainer with 10 ml of erythrocyte lysis buffer and incubated the eluted cells for 5 min at room temperature before washing them with 40 ml 1 × PBS 12, 36. After centrifuging the cell suspension gradients at 500 g for 10 min, we resuspended the cells in 1 × PBS and analyzed them with flow cytometry.

### Flow cytometric analysis

On day 3 after ICH, KikGR and WT mice were analyzed by flow cytometry to investigate intestinal immunocyte trafficking (*n* = 5-6 per group). Cell suspensions from various locations (lesioned brain, meninges, dCLNs, MLNs, Peyer's patches, and peripheral blood) were prepared in 50 µl of FACS buffer (2% FBS, 0.05% NaN3 in PBS) and stained with specific antibodies: APC-Cyanine7 anti-mouse CD45 (103116, Biolegend), APC anti-mouse CD3 (100236, Biolegend), FITC anti-mouse CD4 (100510, Biolegend), Pacific Blue anti-mouse CD8a (100725, Biolegend), PE anti-mouse α4β7 integrin (120606, Biolegend), PE-Cyanine7 anti-mouse CD103 (110910, Biolegend) antibodies. We then examined the samples using a NOVOCYte31310 analyzer (ACEA, USA) for KikGR genotypic cells and a CytoFLEX analyzer (Beackman, USA) for other WT cells. We removed dead cells using a Zombie Aqua fixable viability kit (423101, Biolegend) and analyzed data using FlowJo software (version 10, Tree Star, USA). Isotype control antibodies coupled with appropriate fluorescein were used to establish compensation and gating parameters 9.

### Statistical analysis

The sample sizes were determined based on our previous research 9, 29. We used SPSS 25.0 or GraphPad Prism 8 software to analyze experimental data. The distribution and homogeneity of each dataset were determined using the Shapiro-Wilk test and the variance tests. We present quantitative data as mean ± standard deviation or median with interquartile range. Mortality was evaluated using the chi-square test, and differences between the two groups were assessed using a two-tailed paired t-test if the data were normally distributed or the Mann-Whitney U test if not. For changes between multiple groups, we used a one-way analysis of variance (ANOVA, parametric) followed by Bonferroni correction for multiple comparisons or the Kruskal-Wallis test (nonparametric). To evaluate the differences between treatment groups over time for body weight and rectal temperature, we used repeated measures ANOVA. However, to assess NDS and the corner turn test among multiple groups over time, we employed generalized estimation equations (GEE, nonparametric) followed by a two-tailed paired t-test or the Mann-Whitney U test. *P* < 0.05 was considered statistically significant. See statistics reporting provided in the Supplementary PDF file.

## Results

### Subdiaphragmatic vagotomy (SDV) promotes immunocyte trafficking of MLNs in mice with ICH

**Figure 1:**
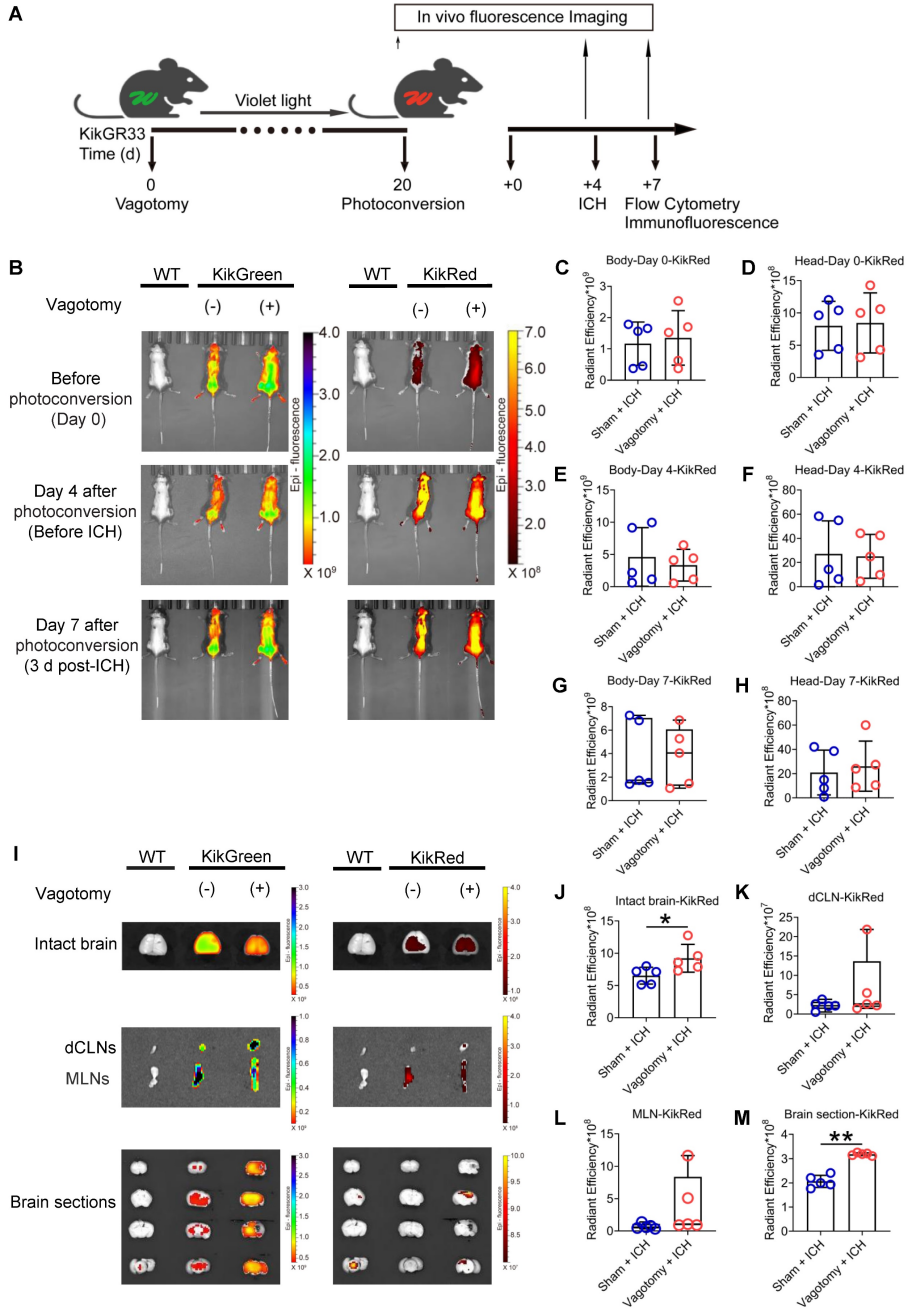
** Dynamic fluorescent changes detected with the *in vivo* imaging system in KikGR mice experienced SDV, photoconversion, and ICH. A)** Workflow for processing KikGR mice. Photoconversion of the MLNs was induced with ultraviolet light irradiation in KikGR mice 20 d after SDV. The ICH model was established 4 d after photoconversion. Fluorescent detection was conducted immediately before photoconversion, immediately before ICH, and 3 d after ICH. **B)** Representative green (KikG) and red (KikR) fluorescent images observed with the *in vivo* imaging system in the heads and bodies of WT mice 3 d after ICH and KikGR mice had previously undergone SDV immediately before photoconversion and 4 d (immediately before ICH) and 7 d (3 d after ICH) after photoconversion. The fluorescent intensity of KikG and KikR in WT mice was set as zero standard. **C-H)** Analysis for the average intensity of red fluorescence (KikR) in the heads and bodies of KikGR mice that had previously undergone a sham vagotomy or SDV at the above 3 time points. *n* = 5 mice per group. The two-tailed Mann-Whitney U test for the red fluorescence (KikR) was performed in the bodies 7 d after photoconversion and the two-tailed paired *t*-test for others. No difference was detected. **I)** Representative fluorescent green (KikG) and red (KikR) images detected with the* in vivo* imaging system in intact isolated brains, dCLNs, MLNs, and brain sections of KikGR mice that had previously undergone a sham vagotomy or SDV 7 d after photoconversion (3 d after ICH). Similar tissues, isolated from WT mice 3 d after ICH, were used as zero controls to detect green (KikG) and red (KikR) fluorescence. **J-M)** Analysis of the mean intensity of red fluorescence (KikR) in the immediately removed brain, dCLNs, MLNs, and brain sections of KikGR mice had previously undergone a sham vagotomy or SDV 7d after photoconversion (3 d post-ICH). *n* = 5 mice per group. The two-tailed paired *t*-test for red fluorescence (KikR) was performed in the intact brain and brain sections, and the Mann-Whitney U test for red fluorescence (KikR) was performed in dCLNs and MLNs. *P < 0.05, **P < 0.01.

Based on a previous study 37, we confirmed the success of SDV by comparing the inhibitory abilities of cholecystokinin-8 (CCK-8) in food intake in wildtype (WT) mice 8 d after sham surgery with those of SDV (Figure S1B). We also tracked intestinal immunocyte migration with an *in vivo* imaging system in KikGR mice that experienced SDV and MLN photoconversion before ICH. The MLN photoconversion was performed 20 d after SDV, followed by ICH induction 4 d later (Figure 1A). Intestinal immunocyte trafficking was detected by monitoring changes in the intensity of green (KikG) and red fluorescence (KikR) in the heads and bodies of KikGR mice immediately before photoconversion or ICH (4 d after photoconversion), as well as 3 d after ICH (7 d after photoconversion). The average intensity of green fluorescence in the heads and bodies of KikGR mice that experienced SDV or sham surgery did not change at any of the above 3-time points (Figures 1B and Figures S2A-F), and the average intensities of red fluorescence did not change immediately before photoconversion or ICH (Figures 1B-F). However, mice that had undergone SDV earlier tended to increase mean intensities of red fluorescence in their heads and bodies 3 d after ICH (Figures 1B, 1G, and 1H). Furthermore, the average intensities of green fluorescence in the intact brain, deep cervical lymph nodes (dCLNs), MLNs, and brain sections were similar in KikGR mice that had undergone SDV previously compared to those who had only experienced a sham vagotomy 3 d post-ICH (Figures 1I and Figures S1G-J). However, previous SDV increased the mean intensities of red fluorescence in intact fresh brains and brain sections but not in dCLNs or MLNs 3 d after ICH (Figures 1I-M).

**Figure 2:**
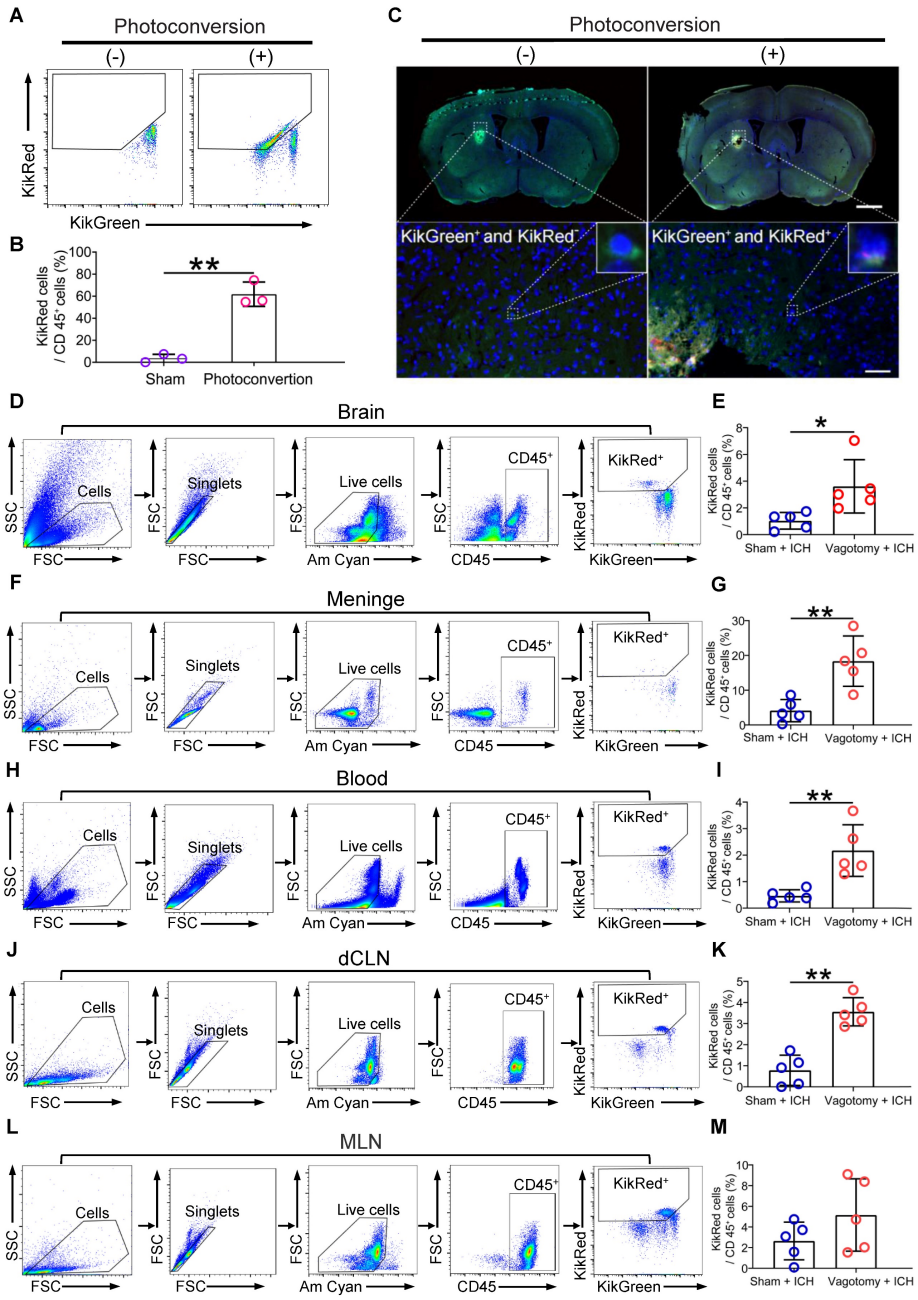
** SDV increases intestinal immunocyte trafficking in KikGR mice with ICH. A)** Representative flow cytometric results for photoconverted immunocytes (CD45^+^KikR^+^ cells) in MLNs of mice 7 d after sham surgery or photoconversion. These mice did not experience SDV or ICH before and after the conversion surgery. **B)** Analysis of photoconverted immunocytes (CD45^+^KikR^+^ cells) in MLNs of mice mentioned above. *n* = 5 mice per group. The two-tailed Mann-Whitney U test was performed. **P < 0.01. **C)** The fluorescence in brain sections from non-photoconverted and photoconverted mice on day 3 after ICH. These mice did not experience SDV but underwent sham conversion surgery or photoconversion 4 d before ICH. The insets in the lower figures show a higher magnification of non-photoconverted (KikG^+^) and photoconverted (KikR^+^) cells. Scale bar of the upper figures = 500 μm. Scale bar of the lower figures = 50 μm. **D, F, H, J, and L**) Representative flow cytometric results for the detection of CD45^+^KikR^+^ cells in brain tissues (D), meninges (F), peripheral blood (H), dCLNs (J), and MLNs (L) 3 d after ICH. These mice experienced SDV 20 d before photoconversion and underwent ICH 4 d after photoconversion. **E, G, I, K, and M)** Analysis for the percentages of red fluorescent cells in CD45^+^ cells in brain tissues (E), meninges (G), peripheral blood (I), dCLNs (K), and MLNs (M) 3 d after ICH. *n* = 5 mice per group. The two-tailed paired t-test was performed. *P < 0.05, **P < 0.01.

The *in vivo* imaging system may be limited to tissue surrounded by bone and cannot identify cell phenotypes *in vivo*. To verify the efficacy of photoconversion of immunocytes in MLNs, we confirmed the presence of photoconverted immunocytes (CD45^+^KikR^+^) with flow cytometry in the MLNs of KikGR mice that underwent photoconvertible surgery or a sham operation without experiencing SDV or ICH (Figures 2A-B). We also tracked the migration of photoconverted cells from the MLNs to the perihematomal regions by detecting green and red fluorescence with a Panoramic Digital Slide Scanner 3 d after ICH (Figure 2C). Our flow cytometric analysis revealed that previous SDV increased the ratio of photoconverted intestinal immunocytes (CD45^+^KikR^+^) to CD45^+^ cells in the hemorrhagic brain, meninges, peripheral blood, and dCLNs, but not in the MLNs 3 d after ICH (Figures 2D-M). Therefore, previous SDV promoted intestinal immunocyte trafficking in ICH.

### SDV exacerbates neuroinflammation, brain injury, and long-term neurologic deficits in mice with ICH

**Figure 3:**
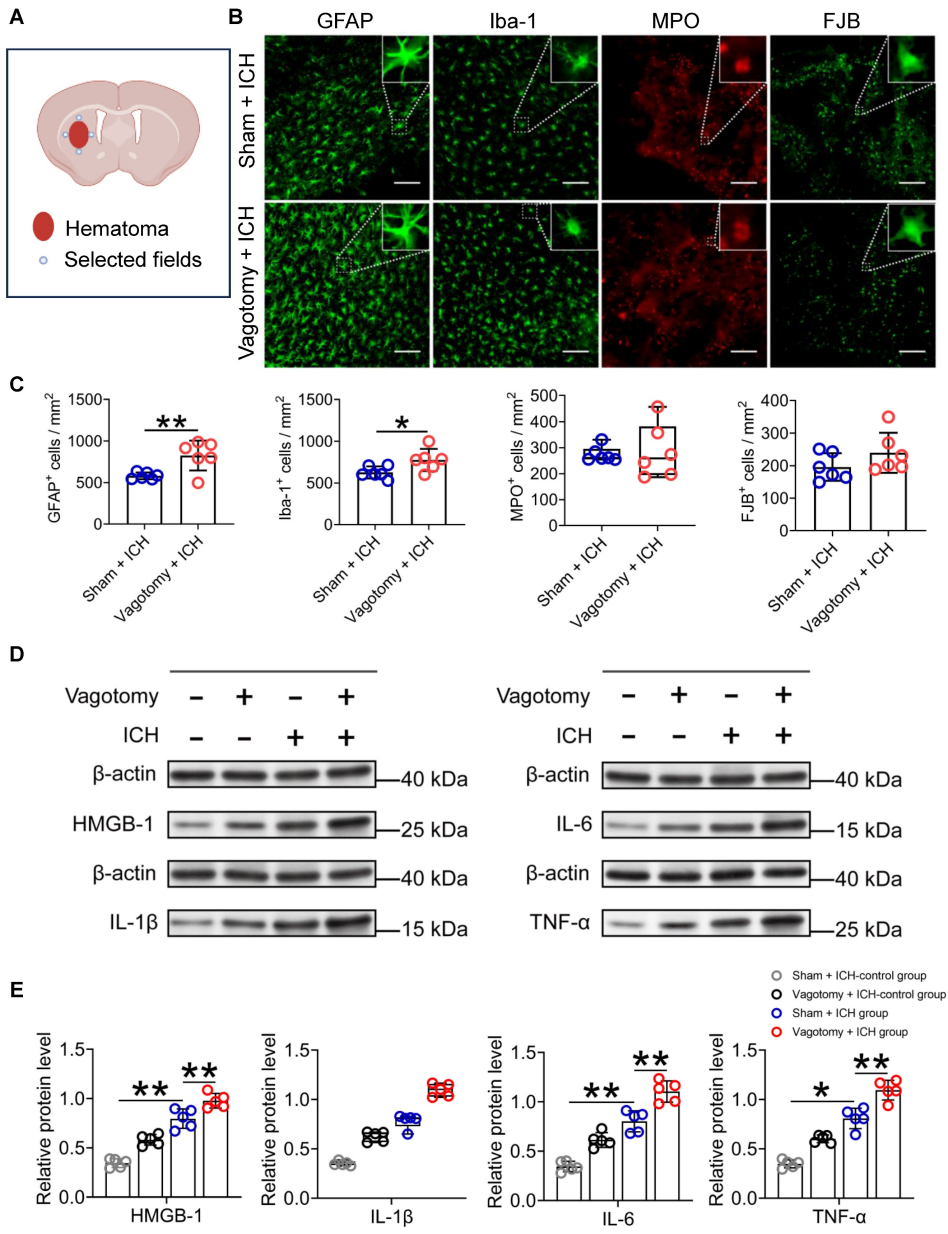
** SDV enhances the cellular and molecular inflammatory response in the hemorrhagic brain. A)** Schematic diagram of the selected fields for the quantification of fluorescent-stained cells. **B)** Representative images of immunofluorescence staining for Iba-1, GFAP, MPO, and FJB in the perihematomal regions 3 d after ICH in WT mice had previously undergone sham vagotomy or SDV. The insets show a higher magnification of immunofluorescence-staining positive cells. Scale bar = 100 μm. **C)** Analysis for microglia/macrophage and astrocytic activation, neutrophil infiltration, and neural degeneration 3 d after ICH. *n* = 6 mice per group. The two-tailed Mann-Whitney U test for MPO and the two-tailed paired t-test for others were performed. *P < 0.05, **P < 0.01. **D)** Representative Western blot bands of HMGB1, IL-1β, IL-6, and TNF-α 1 day after sham operation and hemorrhagic brains of mice that had previously experienced sham vagotomy or SDV earlier. β-actin was used as the loading control. **E)** Quantitative analysis of the expression of pro-inflammatory factors HMGB1, IL-1β, IL-6, and TNF-α in hemorrhagic brains 1 d after ICH. *n* = 5 mice per group. The Kruskal-Wallis test for IL-1β and one-way analysis of variance (ANOVA) with Bonferroni correction for others were performed. *P < 0.05, **P < 0.01.

The infiltration of immunocytes from the periphery plays a crucial role in regulating the innate cerebral inflammatory response, thereby influencing ICH outcomes 2, 38. Using the criteria outlined in the Methods section, we observed that the number of activated microglia/macrophages and astrocytes, respectively, labeled with Iba-1 (ionized calcium-binding protein-1) and GFAP (glial fibrillary acid protein) around the hematoma was significantly higher on day 3 after ICH in mice that underwent SDV than in mice that only had a sham vagotomy (Figure S1E and Figures 3A-C). However, administering SDV 20 d before ICH did not affect the number of MPO (myeloperoxidase)-immunoreactive neutrophils in the hemorrhagic brain (Figures 3B-C). Furthermore, SDV did not significantly increase the number of FJB (Fluoro-Jade B)-positive cells in the perihematomal regions 3 d after ICH (Figures 3B-C).

Proinflammatory cytokines can exacerbate the brain injury caused by ICH 8, 39. We did Western blotting to measure HMGB1, IL-1β, IL-6, and TNF-α protein levels 24 h after ICH (Figure S1E). Our findings indicate that the expression of HMGB1, IL-6, and TNF-α protein increased significantly in the caudate nucleus of mice that underwent ICH and had previously undergone a sham vagotomy, compared to those that underwent a sham vagotomy and a sham ICH surgery (Figures 3D-E). The SDV further increased the expression of these proteins in brain tissues surrounding the hematoma of mice that had undergone SDV 24 h after ICH compared to those with a sham vagotomy (Figures 3D-E). Similar to the above, both ICH and ICH with previous SDV have similar effects on the expression of IL-1β in the perihematomal tissues at the specified time point (Figures 3D-E).

**Figure 4:**
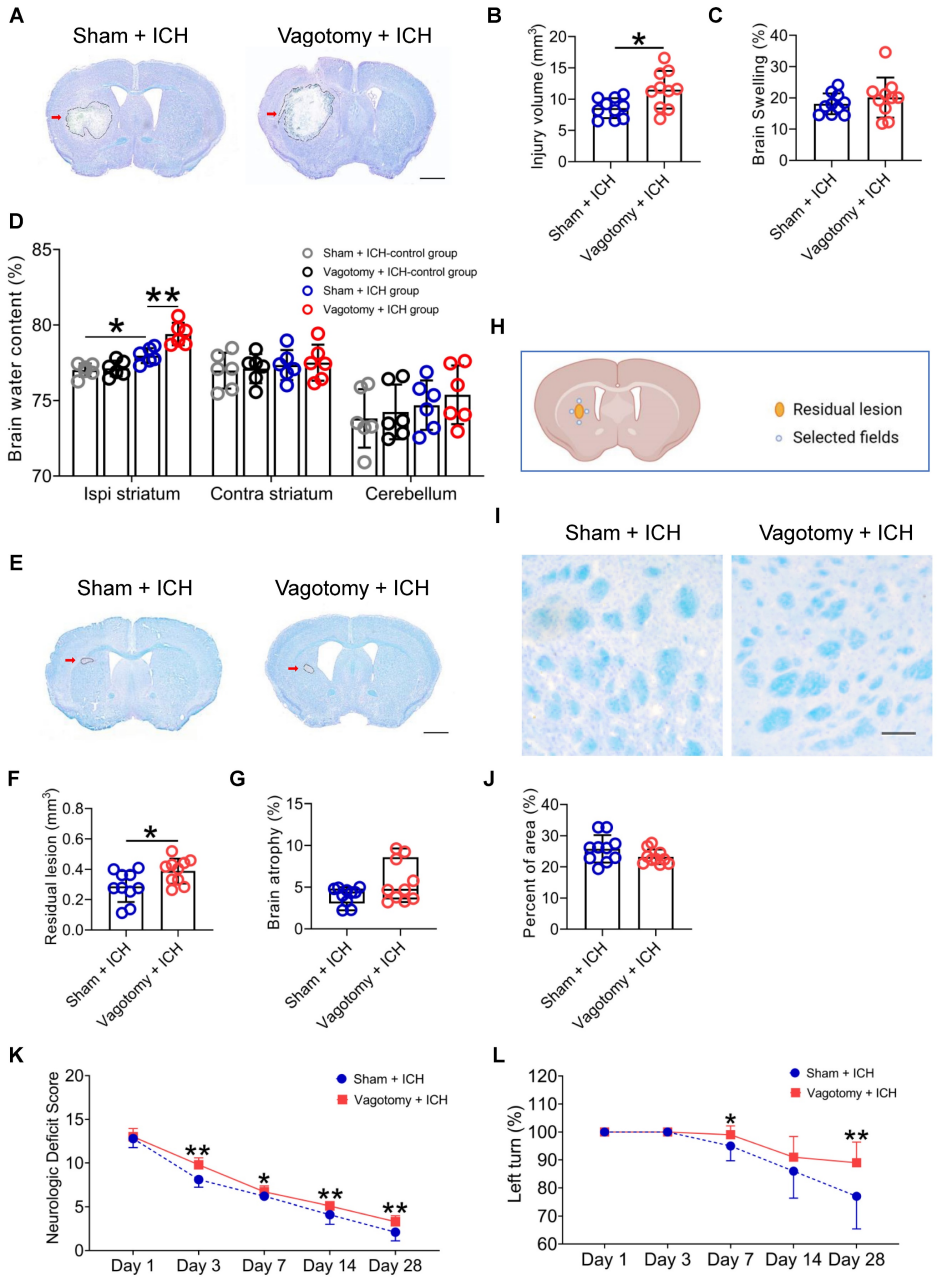
** SDV exacerbates the histopathological and behavioral deficits of mice with ICH. A)** Representative brain sections stained with LFB/CV 3 d after ICH in WT mice that had previously experienced SDV or sham vagotomy. The areas of the lesion lacking staining are circled with a black curve (indicated by a red arrow); scale bar = 500 μm. **B)** Analysis for brain injury volume 3 d after ICH in WT mice that had previously experienced sham vagotomy or SDV. *n* = 10 mice per group. The two-tailed paired t-test was performed, *P < 0.05. **C)** Analysis of brain swelling 3 d after ICH in WT mice that had previously experienced a sham vagotomy or SDV. *n* = 10 mice per group. The two-tailed paired t-test was performed. No difference was found. **D)** Changes in brain water content 3 days after ICH in sham-operated mice and ICH mice that had previously experienced sham vagotomy or SDV. *n* = 6 mice per group. A one-way ANOVA analysis with Bonferroni correction for multiple comparisons was performed, *P < 0.05, **P < 0.01. **E)** Representative residual lesions (indicated by red arrows) in brain sections 28 d after ICH in WT mice that had previously undergone SDV or sham vagotomy. Scale bar = 500 μm. **F)** Analysis of residual lesions in the hemorrhagic brain 28 d after ICH in WT mice that had previously undergone sham vagotomy or SDV. *n* = 10 mice per group. The two-tailed paired t-test was performed, *P < 0.05. **G)** Analysis of brain atrophy in the hemorrhagic brain 28 d after ICH in WT mice that had previously undergone a sham vagotomy or SDV. *n* = 10 mice per group. The Mann-Whitney U test was performed. No significant difference was found.** H)** A schematic diagram of the fields selected for quantifying myelin loss. **I)** Representative images in the perihematomal regions stained with LFB/CV 28 d after ICH in WT mice that had previously undergone sham vagotomy or SDV. Scale bar =100 μm. **J)** Quantitative analysis of white matter damage 28 d after ICH in WT mice that had previously undergone sham vagotomy or SDV. *n* = 10 mice per group. The two-tailed paired t-test was performed. No significant difference was found. **K)** Neurological deficits evaluated with neurologic deficit scores (NDS) throughout the 28-day research process in WT mice that had previously undergone a sham vagotomy or SDV. *n* = 10 mice per group. Generalized estimation equations (GEE) with a two-tailed Mann-Whitney U test were performed at multiple time points. Wald χ^2^ = 61.68 and *P* < 0.001 for the total trend of SDV versus sham vagotomy; *P* < 0.001 on day 3,* P* = 0.036 on day 7,* P* = 0.004 on day 14, and *P* = 0.001 on day 28 for SDV versus sham vagotomy, respectively. **L)** Neurologic deficits were evaluated with the corner turn test (CTT) daily throughout the 28-day research process in WT mice that had previously undergone SDV or sham vagotomy. *n* = 10 mice per group. Generalized estimation equations (GEE) with a two-tailed Mann-Whitney U test were performed at multiple time points. Wald χ^2^ = 11.243 and *P* = 0.010 for the total trend of SDV versus sham vagotomy; *P* = 0.03 on day 7 and *P* = 0.004 on day 28 for SDV versus sham vagotomy.

The prognosis of ICH is based on the severity of the brain injury. We delved into the effects of previous SDV on short- and long-term brain injury severity and long-term neurologic deficits after ICH (Figure S1E). We used LFB/CV staining to measure brain lesion volume and swelling. The results showed that SDV conducted 20 days before ICH significantly increased brain lesion volume but not brain swelling 3 d after ICH (Figures 4A-C). We also used the dry-wet weight method to evaluate the water content in the ipsilateral and contralateral striatum and cerebellum. Our findings indicated that the prior SDV considerably increased the water content in the ipsilateral striatum 3 d after ICH (Figure 4D).

Furthermore, we used LFB/CV or LFB staining to assess long-term brain injury severity. We discovered that previous SDV amplified residual lesion volume, but not brain atrophy or white matter damage, 28 d after ICH (Figures 4E-J). Although previous SDV has few influences on individual NDS tests on days 1, 3, 7, 14, and 28 after ICH (Figures S1E and Figure S3A), mice that underwent SDV earlier had higher NDS than those experiencing sham vagotomy on days 3, 7, 14, and 28 after ICH (Wald χ^2^ = 61.68 and *P* < 0.001; Figure 4K). Mice with prior SDV also showed higher corner turn test scores than those that underwent sham vagotomy on days 7 and 28 after ICH but not on days 1, 3, and 14 (Wald χ^2^ = 11.243 and *P* = 0.010; Figure 4L).

These findings suggest that SDV may enhance neuroinflammation and aggravate ICH prognosis by promoting immunocyte migration from the intestine to the hemorrhagic brain. During the project, no mice died 20 d after SDV or sham vagotomy. However, among the mice that underwent SDV, 11 died after ICH (19.64%, 11/56), while 8 of the mice that underwent a sham vagotomy died after ICH (14.29%, 8/56). Our analysis did not reveal differences in the mortality rate of mice undergoing SDV or sham vagotomy (*P* = 0.45). Our findings also indicate that contrary to the results of one previous study but consistent with another 20, 21, SDV did not affect the rectal temperature or body weight of ICH mice during the 28-day behavioral test (Figures S3B-C).

### The ɑ7nAChR agonists restore intestinal immunocyte homing and retention in ICH mice previously subjected to SDV

To evaluate whether the vagus nerve regulates intestinal immune homeostasis through the cholinergic transmitters and their corresponding receptors, we investigated the effects of ɑ7nAChR agonists on intestinal immunocyte trafficking in ICH mice that had previously undergone SDV (Figure S1E). Employing flow cytometry, we detected the proportions of CD3^+^, CD4^+^, CD8^+^, CD3^+^CD4^+^, and CD3^+^CD8^+^ cells to CD45^high^ or CD45^+^ cells in the hemorrhagic brain, peripheral blood, MLNs, or Peyer's patches 3 d after ICH. We also examined gut-selective integrin expression within them. Figures S4-5 summarized their gating strategies in the organs as mentioned above.

**Figure 5:**
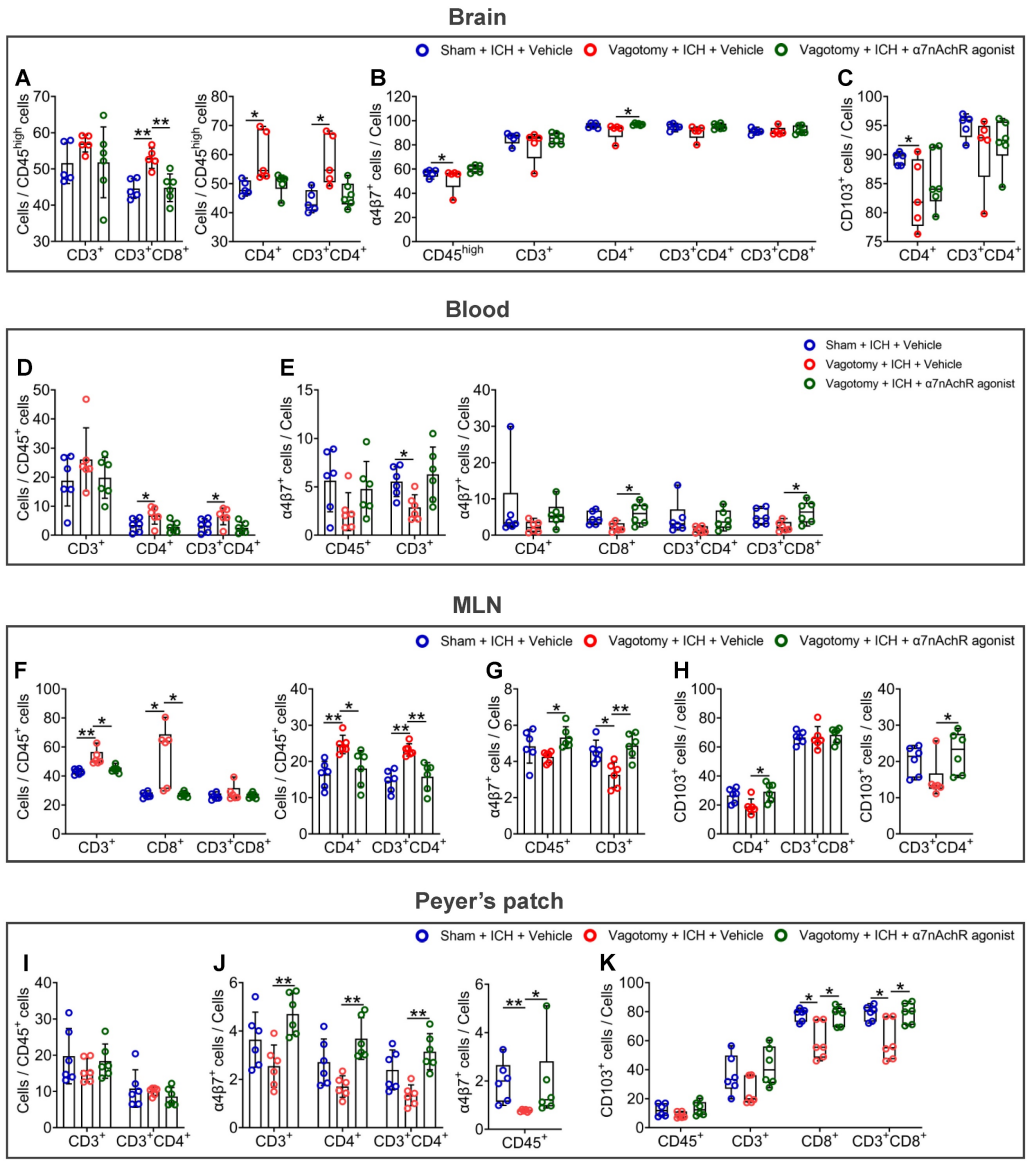
** α7nAChR agonists reverse the homing and retention of intestinal immunocytes of ICH mice that previously underwent SDV. A)** Analysis of the influence of previous SDV and α7nAChR agonist treatment on the infiltration of CD3^+^, CD4^+^, CD3^+^CD4^+^, and CD3^+^CD8^+^ cells quantified by flow cytometry in the brain lesion 3 d after ICH. *n* = 5-6 mice per group. One-way ANOVA followed by Bonferroni correction for CD3^+^ and CD3^+^CD8^+^ cells, while the Kruskal-Wallis test was performed for others. *P < 0.05, **P < 0.01. **B)** Proportional changes of α4β7 integrin-positive CD45^high^, CD3^+^, CD4^+^, CD3^+^CD4^+^, and CD3^+^CD8^+^ cells in the hemorrhagic brain 3 d after ICH.* n* = 5-6 mice per group. The Kruskal-Wallis test was performed. *P < 0.05. **C)** Percentage changes of CD103-positive CD4^+^ and CD3^+^CD4^+^ cells in the hemorrhagic brain 3 d post-ICH. *n* = 5-6 mice per group. The Kruskal-Wallis test was performed. *P < 0.05. **D)** Comparison of the proportions of CD3^+^, CD4^+^, and CD3^+^CD4^+^ cells to CD45^+^ cells in the peripheral blood 3 d after ICH. *n* = 6 mice per group. One-way ANOVA with Bonferroni correction was performed for multiple comparisons. *P < 0.05. **E)** The ratios of α4β7 integrin-positive CD45^+^, CD3^+^, CD4^+^, CD8^+^, CD3^+^CD4^+^, and CD3^+^CD8^+^ cells in the bloodstream 3 d after ICH.* n* = 6 mice per group. One-way ANOVA followed by Bonferroni correction for α4β7 integrin-positive CD45^+^ and CD3^+^ cells, while the Kruskal-Wallis test was performed for others. *P < 0.05. **F)** Analysis of the proportions of CD3^+^, CD4^+^, CD8^+^, CD3^+^CD4^+^, and CD3^+^CD8^+^ cells to CD45^+^ cells in the MLNs 3 d after ICH.* n* = 6 mice per group. The Kruskal-Wallis test for CD3^+^, CD8^+^, and CD3^+^CD8^+^ cells, while one-way ANOVA followed by Bonferroni correction was performed for others. *P < 0.05, **P < 0.01. **G)** Percentage changes of α4β7 integrin-positive CD45^+^ and CD3^+^ cells in the MLNs 3 d after ICH.* n* = 6 mice per group. One-way ANOVA with Bonferroni correction for multiple comparisons was performed. *P < 0.05, **P < 0.01. **H)** Proportional changes of CD103-positive CD4^+^, CD3^+^CD4^+^, and CD3^+^CD8^+^ cells in the MLNs 3 d after ICH.* n* = 6 mice per group. One-way ANOVA followed by Bonferroni correction for CD103-positive CD4^+^ and CD3^+^CD8^+^ cells, while the Kruskal-Wallis test was performed for CD103-positive CD3^+^CD4^+^ cells. *P < 0.05. **I)** Quantification of the percentages of CD3^+^ and CD3^+^CD4^+^ cells to CD45^+^ cells in the Peyer's patch 3 d after ICH.* n* = 6 mice per group. One-way ANOVA with Bonferroni correction was performed for multiple comparisons. No significant difference was found. **J)** Proportional changes of α4β7 integrin-positive CD45^+^, CD3^+^, CD4^+^, and CD3^+^CD4^+^ cells in the Peyer's patch 3 d after ICH.* n* = 6 mice per group. The Kruskal-Wallis test for α4β7 integrin-positive CD45^+^ cells and one-way ANOVA followed by Bonferroni correction was performed for others. *P < 0.05, **P < 0.01. **K)** The percentages of CD103-positive CD45^+^, CD3^+^, CD8^+^, and CD3^+^CD8^+^ cells in the Peyer's patch 3 d after ICH.* n* = 6 mice per group. The Kruskal-Wallis test was performed. *P < 0.05.

The results indicated that previous SDV increased the proportions of CD4^+^, CD3^+^CD4^+^, and CD3^+^CD8^+^ cells to CD45^high^ cells in the hemorrhagic brain 3 d after ICH. However, ɑ7nAChR agonist treatment reversed the above changes in ICH mice (Figure 5A). As for the expression of α4β7 integrin and CD103, known as the αE subunit of αEβ7 integrin, in immunocytes indicated in the hemorrhagic brain, previous SDV reduced the percentages of α4β7 integrin-positive CD45^high^ cells and CD103-positive CD4^+^ cells in the hemorrhagic brain 3 d after ICH. Conversely, ɑ7nAChR agonist treatment after SDV produced contradictory effects on the proportion of α4β7 integrin-positive CD4^+^ cells (Figures 5B-C and Figure S6A).

The alterations in the cell proportions in the peripheral blood (Figure 5D and Figure S6B) closely resemble those detected in the hemorrhagic brain 3 d after ICH. Specifically, after SDV, there was an increase in the proportions of CD4^+^ and CD3^+^CD4^+^ cells to CD45^+^ cells in the bloodstream. Conversely, treatment with ɑ7nAChR agonists after SDV demonstrated contrary effects. Notably, while SDV led to a substantial reduction in the percentages of α4β7 integrin-positive CD45^+^, CD3^+^, CD4^+^, CD8^+^, CD3^+^CD4^+^, and CD3^+^CD8^+^cells, ɑ7nAChR agonist treatment reversed the changes induced by SDV (Figure 5E). This pattern is further evident in the percentage changes of CD103-positive immunocytes, particularly CD103-positive CD3^+^, CD4^+^, and CD3^+^CD4^+^ cells, in the circulation (Figure S6C).

In the context of MLNs, administering a previous SDV increased the ratios of CD3^+^, CD4^+^, CD8^+^, and CD3^+^CD4^+^ cells to CD45^+^ cells. However, the previous SDV combined with ɑ7nAChR agonists reversed these changes 3 d after ICH (Figure 5F). By detecting α4β7 integrin and CD103 expression in the MLNs 3 d after ICH, we observed that administering a previous SDV reduced the proportion of α4β7 integrin-positive CD3^+^ cells. Notably, when combined with ɑ7nAChR agonist treatment, the previous SDV improved the percentages of α4β7 integrin-positive CD45^+^ and CD3^+^ cells (Figure 5G and Figure S6D). Along with ɑ7nAChR agonist treatment, it also increased the percentages of CD103-positive CD4^+^ and CD3^+^CD4^+^ cells (Figure 5H and Figure S6E).

In Peyer's patches, by contrast, previous SDV showed a trend toward decreasing the ratios of CD3^+^ and CD3^+^CD4^+^ cells to CD45^+^ cells and downregulated the percentages of α4β7 integrin-positive CD45^+^, CD3^+^, CD4^+^, and CD3^+^CD4^+^ cells. However, the administration of the previous SDV with ɑ7nAChR agonists reversed the observed changes 3 d after ICH (Figures 5I-J, and Figures S6F-G). While previous SDV reduced the percentage of CD103-positive CD8^+^ and CD3^+^CD8^+^ cell populations in this location, previous SDV combined with ɑ7nAChR agonists also reversed these changes 3 d after ICH (Figure 5K and Figure S6H).

Our observations about the hemorrhagic brain, peripheral blood, MLNs, and Peyer's patches indicate the potential viability of employing an α7nAChR agonist to mitigate the deleterious consequences of previous SDV after ICH. Moreover, the vagus nerve's cholinergic output may serve to impede the migration of immunocytes within the intestine by preserving the expression of α4β7 and αEβ7 integrins after ICH.

### The α7nAChR agonists are ineffective against the stimulatory effects of β7 integrin antagonist etrolizumab on intestinal immunocyte trafficking in mice with ICH

Etrolizumab, as a β7 integrin antagonist, has been developed specifically to impede the effects of α4β7 and αEβ7 integrins on the homing and retention of intestinal immunocytes 19, thereby establishing its potential as a therapeutic agent to treat inflammatory bowel disease 19. To elucidate the impact of vagal innervation on intestinal immunocyte trafficking, we subsequently examined the influence of etrolizumab or α7nAChR agonists combined with etrolizumab on proportional changes in various immunocytes in the hemorrhagic brain, peripheral blood, MLNs, and Peyer's patches 3 d after ICH (Figure S1E). The flow cytometry gating strategies employed to determine the proportions of CD45^+^, CD3^+^, CD4^+^, CD8^+^, CD3^+^CD4^+^, and CD3^+^CD8^+^ cells in the organs above are summarized in Figures S7-8.

**Figure 6:**
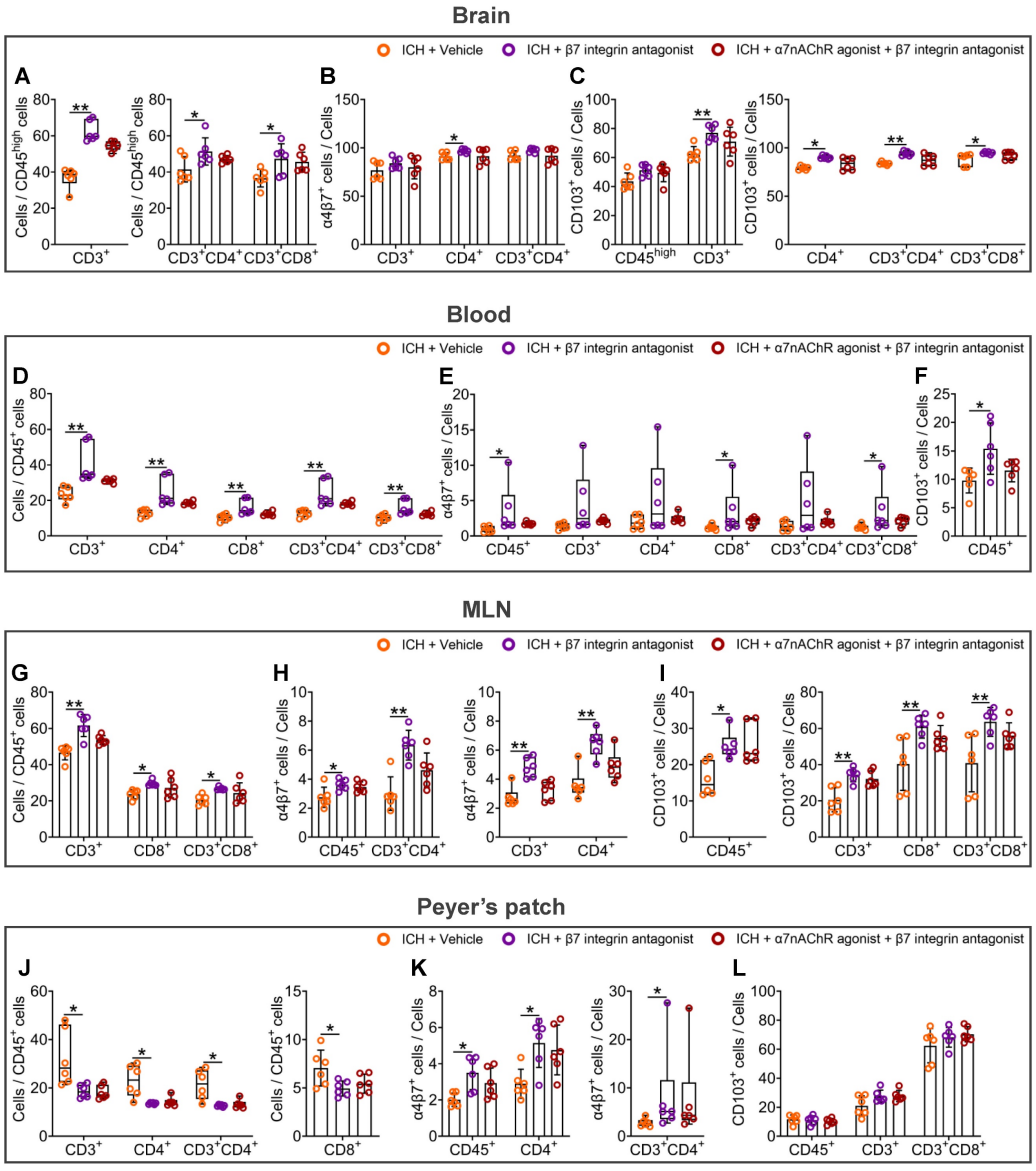
** α7nAChR agonists have no influence on intestinal immunocyte homing and retention enhanced by β7 integrin antagonist etrolizumab in ICH. A)** Evaluation for the influence of β7 integrin antagonist or β7 integrin antagonist with α7nAChR agonist treatment on the infiltration of CD3^+^, CD3^+^CD4^+^, and CD3^+^CD8^+^ cells in the lesioned brain 3 d after ICH. *n* = 6 mice per group. The Kruskal-Wallis test for CD3^+^ cells and one-way ANOVA followed by Bonferroni correction for others were performed. *P < 0.05, **P < 0.01. **B)** Proportional changes of α4β7 integrin-positive CD3^+^, CD4^+^, and CD3^+^CD4^+^ cells in the hemorrhagic brain 3 d after ICH.* n* = 6 mice per group. One-way ANOVA with Bonferroni correction for multiple comparisons was performed. *P < 0.05. **C)** Percentage changes of CD103-positive CD45^high^, CD3^+^, CD4^+^, CD3^+^CD4^+^, and CD3^+^CD8^+^ cells in the hemorrhagic brain 3 d post-ICH. *n* = 6 mice per group. One-way ANOVA with Bonferroni correction for multiple comparisons of CD45^high^ and CD3^+^ cells, while the Kruskal-Wallis test for others was performed. *P < 0.05, **P < 0.01. **D)** Analysis for the proportions of CD3^+^, CD4^+^, CD8^+^, CD3^+^CD4^+^, and CD3^+^CD8^+^ cells to CD45^+^ cells in the bloodstream 3 d after ICH. *n* = 6 mice per group. The Kruskal-Wallis test was performed. **P < 0.01. **E)** The ratio of α4β7 integrin-positive CD45^+^, CD3^+^, CD4^+^, CD8^+^, CD3^+^CD4^+^, and CD3^+^CD8^+^ cells in the periphery 3 d post-ICH.* n* = 6 mice per group. The Kruskal-Wallis test was performed. *P < 0.05. **F)** Percentage change of CD103-positive CD45^+^ cells in the peripheral blood 3 d after ICH. *n* = 6 mice per group. A one-way ANOVA with Bonferroni correction for multiple comparisons was performed. *P < 0.05. **G)** Comparison of the proportions of CD3^+^, CD8^+^, and CD3^+^CD8^+^ cells to CD45^+^ cells in the MLNs 3 d after ICH. *n* = 6 mice per group. One-way ANOVA followed by Bonferroni correction was performed. *P < 0.05, **P < 0.01. **H)** Percentage changes of α4β7 integrin-positive CD45^+^, CD3^+^, CD4^+^, and CD3^+^CD4^+^ cells in the MLNs 3 d after ICH.* n* = 6 mice per group. One-way ANOVA with Bonferroni correction for the proportions of CD45^+^ and CD3^+^CD4^+^ cells, while the Kruskal-Wallis test for others was performed. *P < 0.05, **P < 0.01. **I)** Proportional changes of CD103-positive CD45^+^, CD3^+^, CD8^+^, and CD3^+^CD8^+^ cells in the MLNs 3 d after ICH. *n* = 6 mice per group. The Kruskal-Wallis test for CD103-positive CD45^+^ cells and one-way ANOVA followed by Bonferroni correction for others were performed. *P < 0.05, **P < 0.01. **J)** Quantification of the ratios of CD3^+^, CD4^+^, CD8^+^, and CD3^+^CD4^+^ cells to CD45^+^ cells in the Peyer's patches 3 d after ICH. *n* = 6 mice per group. The Kruskal-Wallis test for CD3^+^, CD4^+^, and CD3^+^CD4^+^ cell percentages and one-way ANOVA with Bonferroni correction for multiple comparisons of CD8^+^ cell percentage were performed. *P < 0.05. **K)** The proportions of α4β7 integrin-positive CD45^+^, CD4^+^, and CD3^+^CD4^+^ cells in the Peyer's patches 3 d after ICH. *n* = 6 mice per group. A one-way ANOVA with Bonferroni correction for multiple comparisons of CD45^+^ and CD4^+^ cells, while the Kruskal-Wallis test for CD3^+^CD4^+^ cells was performed. *P < 0.05. **L)** The percentages of CD103-positive CD45^+^, CD3^+^, and CD3^+^CD8^+^ cells in the Peyer's patches 3 d post-ICH. *n* = 6 mice per group. One-way ANOVA with Bonferroni correction for multiple comparisons. No significant differences were found.

The statistical analysis results indicate that the β7 integrin antagonist, etrolizumab, led to a significant increase in the proportions of CD3^+^, CD3^+^CD4^+^, and CD3^+^CD8^+^ cells compared to CD45^high^ cells in the hemorrhagic brain 3 d after ICH (Figure 6A and Figure S9A). Moreover, α7nAChR agonists did not affect these ratios in the hemorrhagic brain of mice treated with etrolizumab (Figure 6A and Figure S9A). Notably, while etrolizumab elevated the proportions of α4β7 integrin-positive CD4^+^ cells and CD103-positive CD3^+^, CD4^+^, CD3^+^CD4^+^, and CD3^+^CD8^+^ cells in the hemorrhagic brain, α7nAChR agonists also failed to reverse α4β7 integrin and CD103 expression in the immunocytes infiltrated into the brain post-ICH (Figures 6B-C and Figure S9B).

The administration of etrolizumab resulted in a notable increase in the proportions of various immune cell types in the peripheral blood, including CD3^+^, CD4^+^, CD8^+^, CD3^+^CD4^+^, and CD3^+^CD8^+^ cells compared to CD45^+^ cells 3 d after ICH (Figure 6D). Furthermore, there was a heightened proportion of CD45^+^, CD8^+^, and CD3^+^CD8^+^ cells expressing the α4β7 integrin (Figure 6E). There was also an enhanced proportion of CD45^+^ cells expressing CD103 (Figure 6F and Figure S9C). When α7nAChR agonists were employed in combination with etrolizumab, a comparable impact on the immunocyte subpopulations within the peripheral blood was observed, analogous to the effects elicited by etrolizumab alone (Figures 6D-F and Figure S9C).

In the MLNs, etrolizumab treatment exhibited a notable increase in the proportions of CD3^+^, CD8^+^, and CD3^+^CD8^+^ cells compared to CD45^+^ cells 3 d after ICH (Figure 6G and Figure S9D). In addition to the proportions of α4β7 integrin-positive CD45^+^, CD3^+^, CD4^+^, and CD3^+^CD4^+^ cells, it also significantly elevated the percentages of CD103-positive CD45^+^, CD3^+^, CD8^+^, and CD3^+^CD8^+^ cells (Figures 6H-I and Figures S9E-F). Corresponding to the patterns observed in the hemorrhagic brain and peripheral blood, α7nAChR agonists failed to influence the alterations above induced by etrolizumab after ICH (Figures 6G-I and Figures S9D-F).

In Peyer's patches, etrolizumab significantly influenced the proportions of various cellular components after ICH. Specifically, 3 days post-ICH, etrolizumab markedly reduced the proportions of CD3^+^, CD4^+^, CD8^+^, and CD3^+^CD4^+^ cells to CD45^+^ cells (Figure 6J and Figure S9G). Additionally, it elevated the percentages of α4β7 integrin-positive CD45^+^, CD4^+^, and CD3^+^CD4^+^ cells while exhibiting no discernible impact on CD103-positive cellular components in the Peyer's patches at this time point (Figures 6K-L and Figures S9H-I). However, no statistically significant changes in the proportions of the immunocyte above were observed following etrolizumab intervention in the context of ICH when α7nAChR agonists were administered (Figures 6J-L and Figures S9G-I).

The findings in the hemorrhagic brain, peripheral blood, MLNs, and Peyer's patches suggest that the β7 integrin antagonist etrolizumab enhances the migration of intestinal immunocytes after ICH. In this process, α7nAChR agonists cannot abolish the inhibitory effects of β7 integrin antagonist etrolizumab on intestinal immunocyte homing and retention. Thus, α4β7 and CD103 integrins may represent efficacious regulation targets of vagus nerve and its cholinergic transmitters for the trafficking of intestinal immunocytes in ICH.

### The β7 integrin antagonist etrolizumab exacerbates neuroinflammation and hinders functional recovery after ICH

To investigate the potential influence of vagal innervation on intestinal immunocyte trafficking and its impact on neuroinflammation and ICH prognosis, we conducted a study examining the effects of the β7 integrin antagonist etrolizumab on neuroinflammation, short- and long-term brain injury severity, and long-term functional recovery post-ICH.

**Figure 7:**
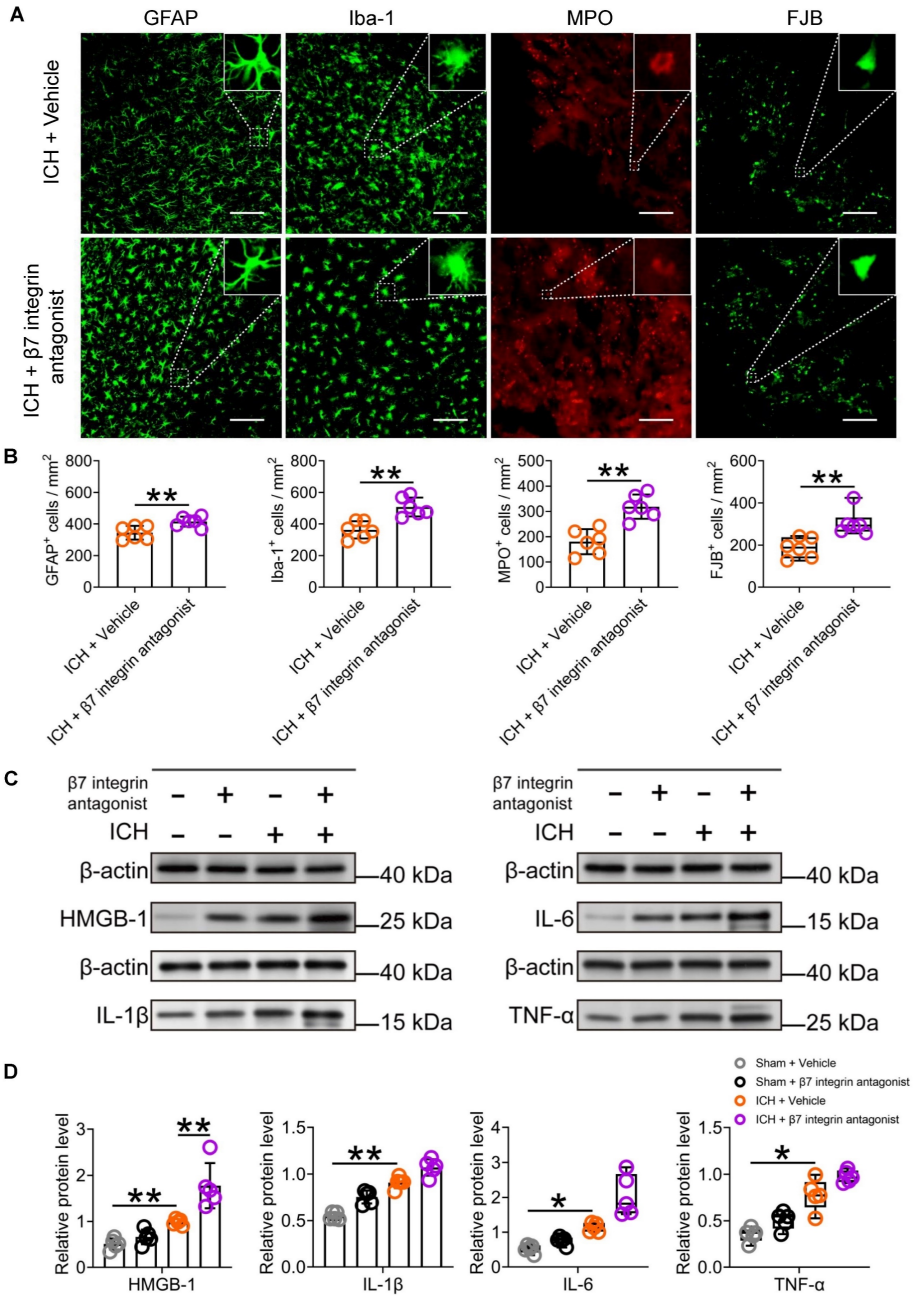
** The β7 integrin antagonist etrolizumab enhances cellular and molecular inflammatory reactions in the lesioned brain after ICH. A)** Immunostaining for GFAP, Iba-1, MPO, and FJB in the perihematomal region 3 d after ICH in mice treated with vehicle or β7 integrin antagonists. The insets show a higher magnification of immunofluorescence-stained cells, Scale bar = 100 μm. **B)** Analysis of microglia/macrophage and astrocytic activation, neutrophil infiltration, and neural degeneration 3 d after ICH. *n* = 6 mice per group. The two-tailed paired t-tests were used for GFAP-, IBA-1- and FJB-positive cells, and the Mann-Whitney U test for MPO-positive cells. **P < 0.01. **C)** Representative Western blot bands of proinflammatory factors HMGB1, IL-1β, IL-6, and TNF-α 3 d after ICH, and β-actin was used as the loading control. **D)** Analysis of the expression of HMGB1, IL-1β, IL-6, and TNF-α in the hemorrhagic brain 1 day after ICH in sham-operated and ICH mice treated with vehicle or β7 integrin antagonist. *n* = 5 mice per group. One-way ANOVA with Bonferroni correction for HMGB1 and IL-1β, and the Kruskal-Wallis test for IL-6 and TNF-α. *P < 0.05, **P < 0.01.

Subsequent analysis of immunofluorescence and Western blotting revealed that the administration of etrolizumab resulted in increased activation of microglia/macrophages and astrocytes, as evidenced by Iba-1 and GFAP staining, surrounding the hematoma 3 d after ICH (Figures 7A-B). This promoted MPO-labelled neutrophil infiltration and exacerbated FJB-positive neuronal degeneration in the perihematomal tissue 3 d after ICH (Figures 7A-7B). Furthermore, it elevated HMGB1 expression and displayed a noticeable tendency to increase IL-1β, IL-6, and TNF-α expression in the ipsilateral striatum 24 h after ICH (Figures 7C-D). These observed changes are likely attributable to the indirect impact of intestinal immunocytes traveling to the brain, a process enhanced by the administration of etrolizumab.

**Figure 8:**
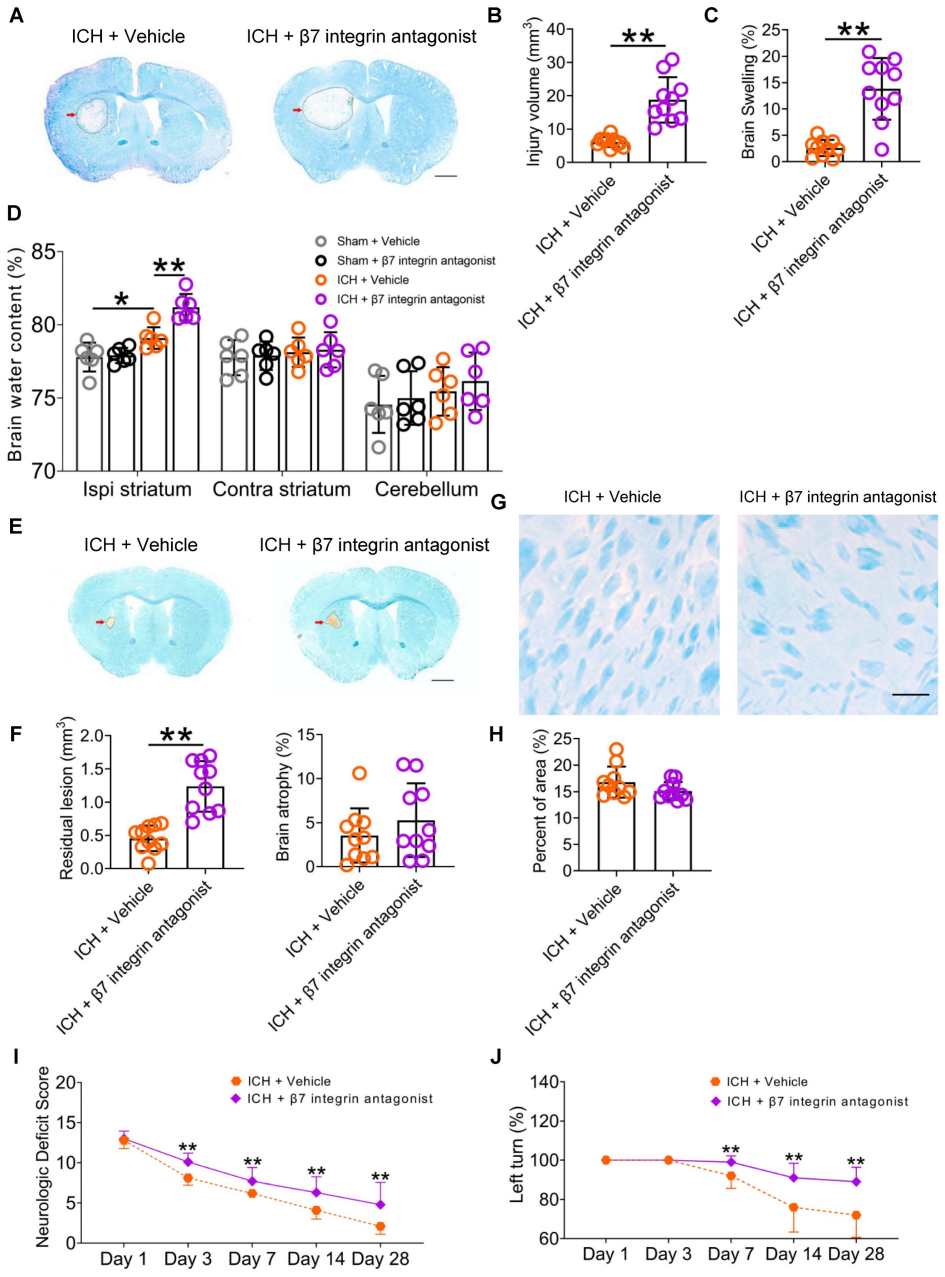
** The β7 integrin antagonist etrolizumab exacerbates brain injury and functional deficits in mice with ICH. A)** Representative brain sections stained with LFB/CV 3 d after ICH. The areas of the lesions lacking staining are circled with a black curve (red arrow indicated); scale bar = 500 μm. **B)** Brain injury volume 3 d after ICH in mice that received vehicle or β7 integrin antagonists. *n* = 10 mice per group. The two-tailed paired t-test was performed. **P < 0.01. **C)** Brain swelling 3 d after ICH in mice that received vehicle or β7 integrin antagonists. *n* = 10 mice per group. The two-tailed paired t-test was performed. **P < 0.01. **D)** Brain water content 3 d after ICH in sham and ICH mice that received vehicle or α7nAChR agonists. *n* = 6 mice per group. One-way analysis of variance followed by Bonferroni correction for multiple comparisons. *P < 0.05, **P < 0.01. **E)** Representative images of brain sections stained with LFB/CV 28 d after ICH. The areas of the lesions are circled with a black curve (the red arrow indicated). Scale bar = 500 μm. **F)** Analysis of residual lesion volume and brain atrophy in ICH mice received vehicle or β7 integrin antagonist. *n* = 10 mice per group. The two-tailed paired t-tests were performed. **P < 0.01. **G)** LFB-stained myelin in the perihematomal region of brain sections 28 d after ICH. Scale bar = 100 μm. **H)** Analysis of white matter damage in the perihematomal regions 28 d after ICH. *n* = 10 mice per group. The two-tailed paired t-test was performed. No significant difference was detected. **I)** Dynamic changes in neurologic deficit scores (NDS) throughout the 28-day research process in WT mice received vehicle or β7 integrin antagonists. *n* = 10 mice per group. Generalized estimation equations (GEE) with a two-tailed paired t-test were performed at multiple time points. Wald χ2 = 33.824, *P* < 0.001 for the total trend of the vehicle versus β7 integrin antagonists; all *P* < 0.01 on days 3, 7, 14, and 28 for the vehicle versus β7 integrin antagonists. **J)** Corner turn test (CTT) throughout the 28-day research process in WT mice received vehicle or β7 integrin antagonists. *n* = 10 mice per group. Generalized estimation equations (GEE) with the two-tailed paired t-test were performed at multiple time points. Wald χ2 = 23.064, *P* < 0.001 for the total trend of the vehicle versus β7 integrin antagonists; all *P* < 0.01 on days 7, 14, and 28 for the vehicle versus β7 integrin antagonists.

Using LFB/CV staining, our study revealed a notable increase in brain injury volume and swelling 3 d post-ICH following the administration of etrolizumab (Figures 8A-C). Additionally, an elevation in brain water content within the ipsilateral striatum was observed (Figure 8D). Despite mitigating residual lesion volume, etrolizumab did not alleviate brain atrophy or white matter damage 28 d post-ICH (Figures 8E-H). Like SDV, etrolizumab treatment worsened total NDS on days 3, 7, 14, 28 after ICH (Wald χ2 = 33.824, *P* < 0.001; Figure 8I). Specifically, administration of etrolizumab exacerbated deficits in circling on days 7 and 28, gait on day 14, body symmetry on day 28, climbing on days 3, 7, 14, and 28, compulsory circling on days 3 and 7, and front limb symmetry on day 28 after ICH Figure S10A). Furthermore, etrolizumab administration increased the left turn ratio on days 7, 14, and 28 after ICH (Wald χ2 = 23.064, *P* < 0.001; Figure 8J).

Our findings suggest that inhibition of intestinal immunocyte trafficking, innervated by the vagus nerve and its cholinergic transmitter receptors, may ameliorate neuroinflammation and enhance functional recovery after ICH. During the experiment, 5 mice died in the vehicle-treated group (7.81%, 5/64) and 3 in the β7 integrin antagonists-treated group after ICH (4.17%, 3/72). Notably, no significant disparity was evident between the two groups (*P* = 0.627). Additionally, the rectal temperature and body weight of the ICH mice remained consistent throughout the 28-day behavioral test period (Figures S10B-C).

## Discussion

The gastrointestinal tract plays an essential role in immune regulation, housing various immunocytes that maintain immune balance post-illness 40, 41. Under stressful conditions, these immunocytes may migrate from the gut to the CNS 12-14. However, the underlying mechanisms and consequences of this migration within the CNS remain unclear. Our study confirms the migration of gastrointestinal immunocytes to the brain and meninges in KikGR mice with ICH. We demonstrate that vagotomy enhances intestinal immunocyte trafficking, resulting in increased neuroinflammation, brain injury, and worsened long-term neurological outcomes following ICH. Conversely, activating α7nAChR reverses the increased immunocyte trafficking and the decreased expression of α4β7 integrin and CD103 induced by vagotomy in ICH mice. Antagonizing β7 integrin with etrolizumab promotes immune cell trafficking within the intestine, leading to exacerbated ICH outcomes. Notably, the stimulatory influence of etrolizumab on intestinal immune cell trafficking is independent of α7nAChR regulation.

As previously reported, αEβ7 integrin facilitates homing of leukocytes to mucosal tissues, while CD103, the αE subunit of αEβ7 integrin, retains these leukocytes within the mucosal tissues 42-44. Our findings highlight the critical role of vagal innervation in suppressing intestinal immunocyte trafficking and improving ICH outcomes. This effect is mediated by upregulating α4β7 and αEβ7 integrin expression in immunocytes via the α7nAChR. These results offer promising avenues for developing novel immunoregulatory therapies that target intestinal immunocyte trafficking by modulating vagus nerve activity, cholinergic transmitters, cholinergic transmitter receptors, or β7 integrin for treating ICH.

The impact of immunocytes present in the bloodstream on hemorrhagic brain injury is widely recognized 2, 8. However, there is a potential for immunocytes to migrate from the gastrointestinal tract to the affected area of the brain after a stroke 12-14, 45. Despite the relatively short lifespan of immune cells, the gastrointestinal tract is a significant reservoir of immune cells that play a vital role in maintaining immune balance within the body 40, 41. Exploring the communication between the gastrointestinal tract and the injured brain after a stroke is essential.

Using photoconvertible KikGR transgenic mice, we monitored the migration of CD45^+^ cells from MLNs to peripheral blood, hemorrhagic brain, meninges, and dCLNs after ICH. Specifically, we accomplished this by converting the color of KikGR in the MLNs from green to red via violet light. Like previous investigations 12, 14, we also observed the immunocyte trafficking from the MLNs of the gastrointestinal tract to the circulation and hemorrhagic brain using flow cytometry after ICH. Furthermore, these findings were further verified with immunofluorescence staining and *in vivo* imaging technologies in this study. The gut-brain axis, an intricate bidirectional communication pathway, integrates neural, hormonal, and immunological signaling between the gut and the brain 14, 46. Although evidence suggests that the intestinal microbiota may mediate this process 12, 13, further elucidation of the precise mechanism is warranted.

Notably, the vagus nerve, as part of the parasympathetic nervous system, also regulates the communication between the gut and the brain 8, 23. Strong evidence has indicated that vagus nerve stimulation (VNS) can significantly alleviate the local and systemic inflammatory response after acute stroke 8, 47. Although its translational value is promising 47, 48, few studies have explored the impact of the vagus nerve on the egress of intestinal immunocytes under stress conditions, including acute stroke. With KikGR mice, we found that SDV promoted the trafficking of immunocytes from the gastrointestinal tract to the circulation and CNS after ICH. In contrast to the immunoinhibitory and neuroprotective effects of VNS previously reported 8, 47, we also observed aggravated neuroinflammation by SDV and worsening of long-term neurologic deficits in WT mice with acute stroke. Thus, this study provided a valuable strategy for regulating intestinal immunocyte trafficking after ICH.

The integrin family members, α4β7 and αEβ7 (CD103) integrins are extensively expressed in immunocytes 49, 50. Functionally, they serve as gut-selective integrin to retain lymphocytes in mucosal tissues by binding to the mucosal address cellular adhesion molecule 1 (MAdCAM-1) and E-cadherin, respectively 19, 49, 51. With WT mice, our analysis revealed that the prior SDV decreased the proportions of α4β7 integrin- or CD103-positive immunocyte subpopulations in the hemorrhagic brain, peripheral blood, MLNs, and Peyer's patches after ICH. However, administrating α7nAChR agonists after SDV reversed these changes. This suggests that SDV and α7nAChR agonists may have contradictory effects on the egress of intestinal immunocytes in ICH. Vagal innervation could have significant implications in promoting immunocyte homing and retention in the intestinal tract by increasing α4β7 and αEβ7 integrin expression through the activation of α7nAChR after ICH.

As an anti-β7 integrin monoclonal antibody, etrolizumab functions to block the activity of integrin family members containing the β7 subunit, notably the α4β7 and αEβ7 integrins 52. Its primary intended application is directed towards treating inflammatory bowel disease, aiming to facilitate the egress of intestinal immunocytes 19. Like SDV, etrolizumab also fosters intestinal lymphocyte migration from the intestinal tract to the peripheral blood and damaged brain of WT mice with ICH. Contradictory to SDV, it increases the proportions of α4β7 integrin- or CD103-positive immunocyte subpopulations in the hemorrhagic brain, peripheral blood, MLNs, and Peyer's patches after ICH. Additionally, it appears that the administration of α7nAChR agonists does not influence the above changes in ICH mice undergoing etrolizumab treatment. In alignment with the adverse impact of etrolizumab on neuroinflammation and functional outcomes highlighted in this study, we further elucidated that vagal innervation restrains intestinal immunocyte trafficking through cholinergic transmitters-mediated α4β7 and αEβ7 integrin expression in ICH. Furthermore, we substantiated that vagus nerve-mediated intestinal immunocyte trafficking holds pivotal significance in the context of ICH.

The α4β7 and αEβ7 integrins are heterodimers composed of the α4 integrin, αE integrin (CD103), and β7 integrin subunits. Studies have shown that α4 integrin expression is upregulated in lymphocytes infiltrating the injured brain following stroke 53, 54. Blocking α4 integrin with specific antibodies may alleviate brain injury by inhibiting leukocyte recruitment after a stroke, including ICH 53, 54. However, our findings regarding the expression and efficacy of α4β7 integrin in the immunocytes infiltrating the hemorrhagic brain contradict those reported for α4 integrin. As α4 integrin forms heterodimers with β1 or β7 integrins, the observed discrepancies may be attributed to the differential expression of α4β1 and α4β7 integrins in various immune cell types. There is evidence that α4β1 is expressed on leukocytes and microglia, whereas α4β7 is found on gut-homing T cells and specific vascular endothelial cells 49, 53. When combining this with the preferential expression of CD103, the αE integrin subunit of αEβ7 integrin, in intestinal intraepithelial lymphocytes and the established role of CD103 in retaining intestinal lymphocytes 49, 50, the observed changes in α4β7 and CD103 integrin expression in this study likely reflect the impact of the vagal cholinergic nerve system on the migration and retention of intestinal immunocytes. These insights offer valuable guidance for future research and clinical applications.

Although vagal innervation was demonstrated to be crucial in limiting intestinal immunocyte migration, this regulation may not be present in the MLNs of mice with ICH. Notably, our study reveals a discernible increase in the mean intensity of red fluorescence (KikR) in the MLNs of KikGR mice following SDV in the acute phase of ICH. Furthermore, prior SDV and etrolizumab treatment led to an elevated proportion of lymphocytes in the MLNs of ICH-afflicted WT mice, contrasting with the observed reduction in the Peyer's patches. Given existing evidence indicating the migration of lymphocytes through intestinal epithelium or from MLNs to Peyer's patches under stressful conditions 19, it is conceivable that vagal innervation may facilitate the migration of intestinal lymphocytes to Peyer's patches via α7nAChR after ICH. These findings open up a world of potential for further research and understanding.

Aside from neuroinflammation, a contrasting immune response occurs in the peripheral blood after stroke, potentially exacerbating the individual's susceptibility to infection and negatively impacting the stroke prognosis 8, 55. The potential exacerbation of systemic immunosuppression through the cholinergic nerve system-mediated immunocyte homing is a critical area that remains unexplored and could significantly impact patient care following ICH. Furthermore, while vagus nerve activity may influence the intestinal flora to regulate the movement of intestinal immunocytes to the brain 12, the potential regulation of α4β7 and αEβ7 integrin expression by the intestinal flora and its subsequent influence on intestinal immunocyte movement is a matter of immediate concern that warrants further investigation. Additionally, there is a need to explore whether the vagus nerve mediates α4β7 and αEβ7 integrin expression in regulatory T (Treg) cells and regulates the migration of intestinal Treg cells, given the contribution of Tregs to immune tolerance in ICH and its potential impact on patient care 8.

In conclusion, our study represents a potential breakthrough: immune cells may migrate from the gastrointestinal tract to the brain during ICH. This discovery and the identification of the vagus nerve and its cholinergic transmitters as potential regulators of immune cell mobilization open up exciting possibilities. By modulating the expression of α4β7 and αEβ7 integrins in these cells, we may be able to control this migration. We have validated that the infiltration of intestinal immune cells into the hemorrhagic brain markedly contributes to neuroinflammation and brain damage after ICH. These findings not only deepen our understanding but also pave the way for developing novel immunomodulatory therapies targeting the trafficking of intestinal immune cells. The potential impact of this research on future medical treatments is exciting and significant.

## Supplementary Material

Supplementary figures.

## Abbreviations

ICH: Intracerebral hemorrhage
SDV: Subdiaphragmatic vagotomy
α7nAChR: α7 nicotinic acetylcholine receptors
KikGR: Kikume Green-Red
MLNs: Mesenteric lymph nodes
LFB: Luxol fast blue
GFAP: Glial fibrillary acid protein
Iba-1: Ionic calcium-binding adapter molecule 1
MPO: Myeloperoxidase
FJB: Fluorescent Jade B
NDS: Neurologic deficit score
CTT: Corner turn test
MAdCAM-1: Address cellular adhesion molecule 1
VNS: Vagus nerve stimulation

## References

[1] Greenberg SM, Ziai WC, Cordonnier C, Dowlatshahi D, Francis B, Goldstein JN (2022). 2022 Guideline for the Management of Patients With Spontaneous Intracerebral Hemorrhage: A Guideline From the American Heart Association/American Stroke Association. Stroke.

[2] Liu Y, Chen S, Liu S, Wallace KL, Zille M, Zhang J (2023). T-cell receptor signaling modulated by the co-receptors: Potential targets for stroke treatment. Pharmacol Res.

[3] Magid-Bernstein J, Girard R, Polster S, Srinath A, Romanos S, Awad IA (2022). Cerebral Hemorrhage: Pathophysiology, Treatment, and Future Directions. Circ Res.

[4] Ren H, Han R, Chen X, Liu X, Wan J, Wang L (2020). Potential therapeutic targets for intracerebral hemorrhage-associated inflammation: An update. J Cereb Blood Flow Metab.

[5] Lan X, Han X, Li Q, Yang QW, Wang J (2017). Modulators of microglial activation and polarization after intracerebral haemorrhage. Nat Rev Neurol.

[6] Xue M, Yong VW (2020). Neuroinflammation in intracerebral haemorrhage: immunotherapies with potential for translation. Lancet Neurol.

[7] Shi SX, Xiu Y, Li Y, Yuan M, Shi K, Liu Q (2023). CD4(+) T cells aggravate hemorrhagic brain injury. Sci Adv.

[8] Zhu L, Huang L, Le A, Wang TJ, Zhang J, Chen X (2022). Interactions between the Autonomic Nervous System and the Immune System after Stroke. Compr Physiol.

[9] Zhang Z, Li Y, Shi J, Zhu L, Dai Y, Fu P (2023). Lymphocyte-Related Immunomodulatory Therapy with Siponimod (BAF-312) Improves Outcomes in Mice with Acute Intracerebral Hemorrhage. Aging Dis.

[10] Fu Y, Hao J, Zhang N, Ren L, Sun N, Li YJ (2014). Fingolimod for the treatment of intracerebral hemorrhage: a 2-arm proof-of-concept study. JAMA Neurol.

[11] Huang Y, Zhao M, Chen X, Zhang R, Le A, Hong M (2023). Tryptophan Metabolism in Central Nervous System Diseases: Pathophysiology and Potential Therapeutic Strategies. Aging Dis.

[12] Benakis C, Brea D, Caballero S, Faraco G, Moore J, Murphy M (2016). Commensal microbiota affects ischemic stroke outcome by regulating intestinal gammadelta T cells. Nat Med.

[13] Bostick JW, Schonhoff AM, Mazmanian SK (2022). Gut microbiome-mediated regulation of neuroinflammation. Curr Opin Immunol.

[14] Brea D, Poon C, Benakis C, Lubitz G, Murphy M, Iadecola C (2021). Stroke affects intestinal immune cell trafficking to the central nervous system. Brain Behav Immun.

[15] Teratani T, Mikami Y, Nakamoto N, Suzuki T, Harada Y, Okabayashi K (2020). The liver-brain-gut neural arc maintains the T(reg) cell niche in the gut. Nature.

[16] Matteoli G, Boeckxstaens GE (2013). The vagal innervation of the gut and immune homeostasis. Gut.

[17] Shand FH, Ueha S, Otsuji M, Koid SS, Shichino S, Tsukui T (2014). Tracking of intertissue migration reveals the origins of tumor-infiltrating monocytes. Proc Natl Acad Sci U S A.

[18] Tomura M (2023). In Vivo Tracking of Dendritic Cell Migration. Methods Mol Biol.

[19] Dai B, Hackney JA, Ichikawa R, Nguyen A, Elstrott J, Orozco LD (2021). Dual targeting of lymphocyte homing and retention through alpha4beta7 and alphaEbeta7 inhibition in inflammatory bowel disease. Cell Rep Med.

[20] Kim S, Kwon SH, Kam TI, Panicker N, Karuppagounder SS, Lee S (2019). Transneuronal Propagation of Pathologic alpha-Synuclein from the Gut to the Brain Models Parkinson's Disease. Neuron.

[21] Ghia JE, Blennerhassett P, Kumar-Ondiveeran H, Verdu EF, Collins SM (2006). The vagus nerve: a tonic inhibitory influence associated with inflammatory bowel disease in a murine model. Gastroenterology.

[22] Xue R, Wan Y, Sun X, Zhang X, Gao W, Wu W (2019). Nicotinic Mitigation of Neuroinflammation and Oxidative Stress After Chronic Sleep Deprivation. Front Immunol.

[23] Wu Y, Zhang Y, Xie B, Abdelgawad A, Chen X, Han M (2021). RhANP attenuates endotoxin-derived cognitive dysfunction through subdiaphragmatic vagus nerve-mediated gut microbiota-brain axis. J Neuroinflammation.

[24] Pu Y, Tan Y, Qu Y, Chang L, Wang S, Wei Y (2021). A role of the subdiaphragmatic vagus nerve in depression-like phenotypes in mice after fecal microbiota transplantation from Chrna7 knock-out mice with depression-like phenotypes. Brain Behav Immun.

[25] Sanchez MR, Wang Y, Cho TS, Schnapp WI, Schmit MB, Fang C (2022). Dissecting a disynaptic central amygdala-parasubthalamic nucleus neural circuit that mediates cholecystokinin-induced eating suppression. Mol Metab.

[26] Li Q, Lan X, Han X, Durham F, Wan J, Weiland A (2021). Microglia-derived interleukin-10 accelerates post-intracerebral hemorrhage hematoma clearance by regulating CD36. Brain Behav Immun.

[27] Li Q, Han X, Lan X, Gao Y, Wan J, Durham F (2017). Inhibition of neuronal ferroptosis protects hemorrhagic brain. JCI Insight.

[28] Bobinger T, Manaenko A, Burkardt P, Beuscher V, Sprugel MI, Roeder SS (2019). Siponimod (BAF-312) Attenuates Perihemorrhagic Edema And Improves Survival in Experimental Intracerebral Hemorrhage. Stroke.

[29] Zhang Z, Song Y, Zhang Z, Li D, Zhu H, Liang R (2017). Distinct role of heme oxygenase-1 in early- and late-stage intracerebral hemorrhage in 12-month-old mice. J Cereb Blood Flow Metab.

[30] Rodemerk J, Junker A, Chen B, Pierscianek D, Dammann P, Darkwah Oppong M (2020). Pathophysiology of Intracranial Aneurysms: COX-2 Expression, Iron Deposition in Aneurysm Wall, and Correlation With Magnetic Resonance Imaging. Stroke.

[31] Zhu J, Liu B, Wang Z, Wang D, Ni H, Zhang L (2019). Exosomes from nicotine-stimulated macrophages accelerate atherosclerosis through miR-21-3p/PTEN-mediated VSMC migration and proliferation. Theranostics.

[32] Zhang Q, Zhang J, Wang P, Zhu G, Jin G, Liu F (2022). Glioma-associated mesenchymal stem cells-mediated PD-L1 expression is attenuated by Ad5-Ki67/IL-15 in GBM treatment. Stem Cell Res Ther.

[33] Nowotschin S, Hadjantonakis AK (2009). Use of KikGR a photoconvertible green-to-red fluorescent protein for cell labeling and lineage analysis in ES cells and mouse embryos. BMC Dev Biol.

[34] Masuda T, Sankowski R, Staszewski O, Bottcher C, Amann L, Sagar (2019). Spatial and temporal heterogeneity of mouse and human microglia at single-cell resolution. Nature.

[35] Louveau A, Smirnov I, Keyes TJ, Eccles JD, Rouhani SJ, Peske JD (2015). Structural and functional features of central nervous system lymphatic vessels. Nature.

[36] Jing W, Chen J, Cai Y, Chen Y, Schroeder JA, Johnson BD (2019). Induction of activated T follicular helper cells is critical for anti-FVIII inhibitor development in hemophilia A mice. Blood Adv.

[37] Ghia JE, Blennerhassett P, Collins SM (2007). Vagus nerve integrity and experimental colitis. Am J Physiol Gastrointest Liver Physiol.

[38] Zhu L, Shang J, Li Y, Zhang Z, Fu P, Zong Y (2024). Toll-Like Receptors Mediate Opposing Dendritic Cell Effects on Treg/Th17 Balance in Mice With Intracerebral Hemorrhage. Stroke.

[39] Zhu H, Wang Z, Yu J, Yang X, He F, Liu Z (2019). Role and mechanisms of cytokines in the secondary brain injury after intracerebral hemorrhage. Prog Neurobiol.

[40] Spencer J, Bemark M (2023). Human intestinal B cells in inflammatory diseases. Nat Rev Gastroenterol Hepatol.

[41] Carloni S, Rescigno M (2022). Unveiling the gut-brain axis: structural and functional analogies between the gut and the choroid plexus vascular and immune barriers. Semin Immunopathol.

[42] Lamb CA, O'Byrne S, Keir ME, Butcher EC (2018). Gut-Selective Integrin-Targeted Therapies for Inflammatory Bowel Disease. J Crohns Colitis.

[43] Zundler S, Becker E, Schulze LL, Neurath MF (2019). Immune cell trafficking and retention in inflammatory bowel disease: mechanistic insights and therapeutic advances. Gut.

[44] Schleier L, Wiendl M, Heidbreder K, Binder MT, Atreya R, Rath T (2020). Non-classical monocyte homing to the gut via alpha4beta7 integrin mediates macrophage-dependent intestinal wound healing. Gut.

[45] Hooper LV, Littman DR, Macpherson AJ (2012). Interactions between the microbiota and the immune system. Science.

[46] Carloni S, Rescigno M (2023). The gut-brain vascular axis in neuroinflammation. Semin Immunol.

[47] Ward N (2023). The prospects for poststroke neural repair with vagal nerve stimulation. J Neurol Neurosurg Psychiatry.

[48] Slomski A (2021). Vagus Nerve Stimulation Restores Arm Function Years After Stroke. JAMA.

[49] Fidelle M, Rauber C, Alves Costa Silva C, Tian AL, Lahmar I, de La Varende AM (2023). A microbiota-modulated checkpoint directs immunosuppressive intestinal T cells into cancers. Science.

[50] Keir ME, Fuh F, Ichikawa R, Acres M, Hackney JA, Hulme G (2021). Regulation and Role of alphaE Integrin and Gut Homing Integrins in Migration and Retention of Intestinal Lymphocytes during Inflammatory Bowel Disease. J Immunol.

[51] Zundler S, Schillinger D, Fischer A, Atreya R, Lopez-Posadas R, Watson A (2017). Blockade of alphaEbeta7 integrin suppresses accumulation of CD8(+) and Th9 lymphocytes from patients with IBD in the inflamed gut in vivo. Gut.

[52] Sommer K, Heidbreder K, Kreiss L, Dedden M, Paap EM, Wiendl M (2023). Anti-beta7 integrin treatment impedes the recruitment on non-classical monocytes to the gut and delays macrophage-mediated intestinal wound healing. Clin Transl Med.

[53] Hammond MD, Ambler WG, Ai Y, Sansing LH (2014). alpha4 integrin is a regulator of leukocyte recruitment after experimental intracerebral hemorrhage. Stroke.

[54] Liesz A, Zhou W, Mracsko E, Karcher S, Bauer H, Schwarting S (2011). Inhibition of lymphocyte trafficking shields the brain against deleterious neuroinflammation after stroke. Brain.

[55] Zeng Y, Nie K, Wallace KL, Li F, Zhang J, Li C (2023). Premorbid Use of Beta-Blockers or Angiotensin-Converting Enzyme Inhibitors/Angiotensin Receptor Blockers in Patients with Acute Ischemic Stroke. Oxidative Medicine and Cellular Longevity.

