# Supplementary material for: Vagal innervation limits brain injury by inhibiting gut-selective integrin-mediated intestinal immunocyte migration in intracerebral hemorrhage
Source: Theranostics. 2024 Oct 28;14(19):7383–404. doi: 10.7150/thno.101680 (PMC11626938; doi:10.7150/thno.101680)
Supplement: Supplementary file 1 — Supplementary figures. [file thnov14p7383s1.pdf]

Supplementary materials

Vagal innervation limits brain injury by inhibiting gut-selective integrin-mediated intestinal immunocyte migration in intracerebral hemorrhage

Peiji Fu[1,2,3#], Yan Zong[1,2,3#], Yinming Dai[1,2#], Li Zhu[1,2], Shuai Chen[1,2,3], Yousef Rastegar-Kashkooli[4], Junmin Wang[4*], Jiewen Zhang[1*], Jian Wang[4*], Chao Jiang[1,2,3*]

[1]Department of Neurology, The People's Hospital of Zhengzhou University & Henan Provincial People's Hospital, 450003, Zhengzhou, P. R. China; [2]Department of Neurology, The Fifth Affiliated Hospital of Zhengzhou University, 450052, Zhengzhou, P. R. China; [3]The Laboratory of Cerebrovascular Diseases and Neuroimmunology, The Fifth Affiliated Hospital of Zhengzhou University, 450052, Zhengzhou, P. R. China; [4]Department of Human Anatomy, School of Basic Medical Sciences, Zhengzhou University, 450001, Zhengzhou, P. R. China.

Supplementary Figures and legends

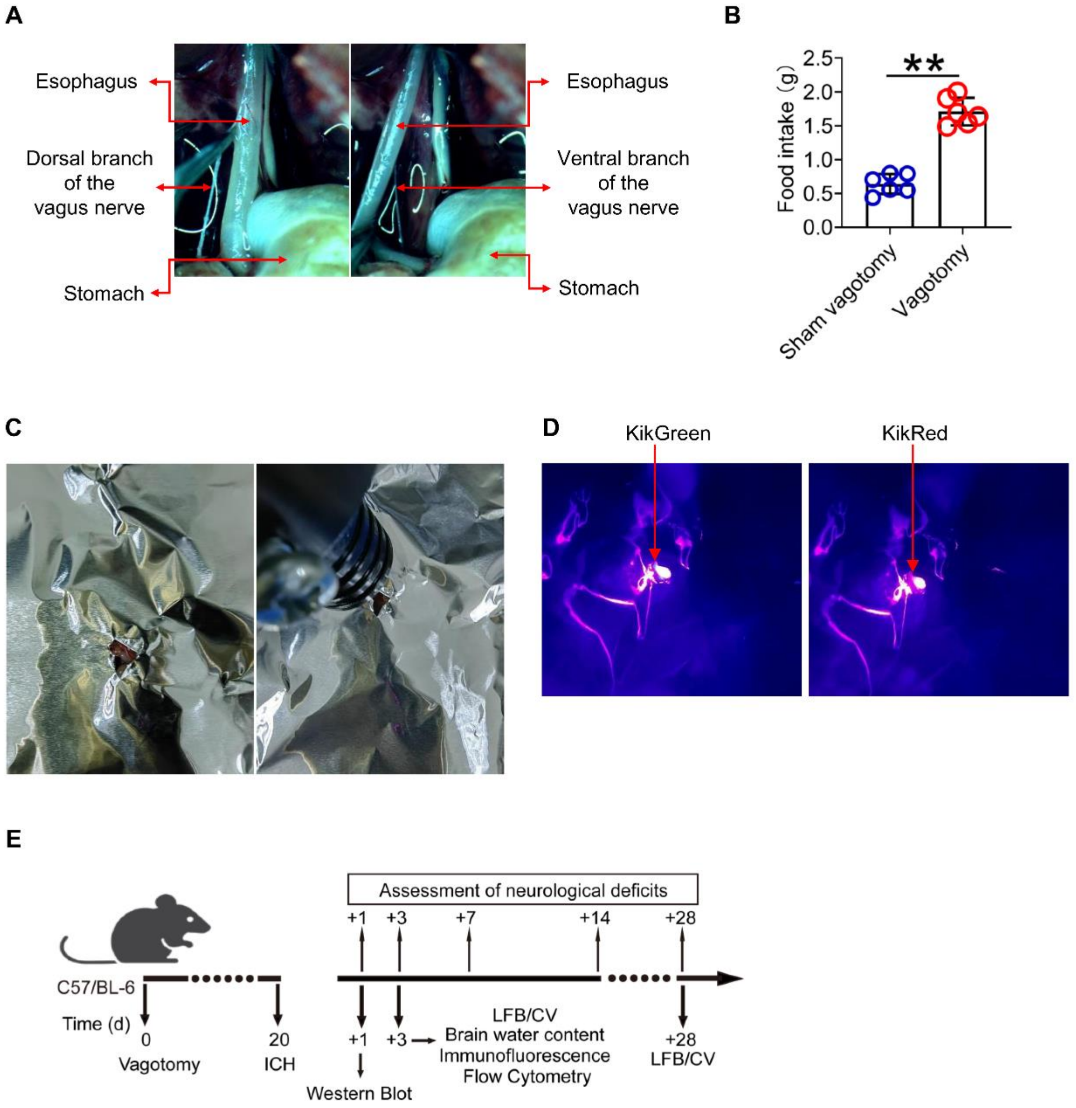

Figure S1. Establishment of a subdiaphragmatic vagotomy (SDV) mouse model and photoconversion of KikGR mice.

A) To establish the mouse subdiaphragmatic vagotomy (SDV), we isolated the ventral and dorsal branches of the vagus nerve. **B)** After SDV, 24-h food intake increased on day 8 compared to sham vagotomy, as determined by the two-tailed paired t-test. n = 5 mice per group. $^{**}P < 0.01$. **C)** During the photoconversion process in KikGR mice, we exposed the MLNs while covering

the surrounding tissues with aluminum foil to prevent additional irradiation. **D)** The MLNs showed green fluorescence (KikG) before and red fluorescence (KikR) after photoconversion. **E)** We created a schematic diagram to illustrate the SDV research in WT mice conducted in this study. We induced ICH in WT mice 20 d after SDV; we then evaluated the neuroinflammation, intestinal immunocyte trafficking, brain injury severity, and neurologic deficits in WT mice that underwent SDV.

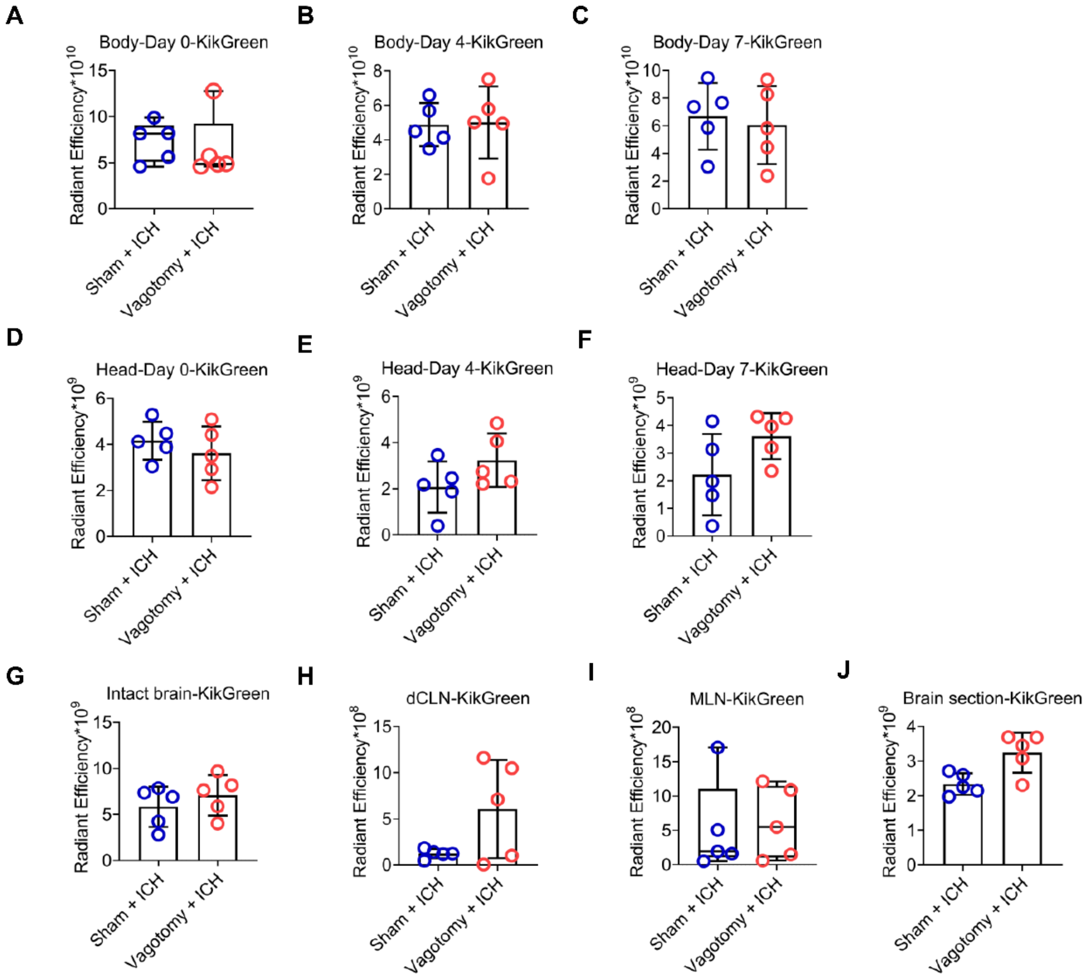

Figure S2. Dynamic changes in green fluorescence detected with an *in vivo* imaging system in KikGR mice experienced SDV, photoconversion, and ICH.

A-C) We measured the green (KikG) fluorescence intensity of KikGR mice in various parts of their bodies at different time points. The first measurements occurred before photoconversion, immediately before ICH (4 d after photoconversion), and 3 d after ICH (7 d after photoconversion). *n* = 5 mice per group. **D-F)** The 2nd set of measurements took place on the heads of the same mice during the same time points. **G-J)** We took the 3rd set of measurements in the freshly isolated brain, dCLNs, MLNs, and brain sections of the same mice

3 d after ICH. *n*=5 mice per group. The average intensity of green (KikG) fluorescence was calculated and analyzed for statistical significance using a two-tailed paired t-test or a two-tailed Mann-Whitney U test, as appropriate. The results indicated no statistical difference in the green (KikG) fluorescence intensity between the various time points or body parts being measured.

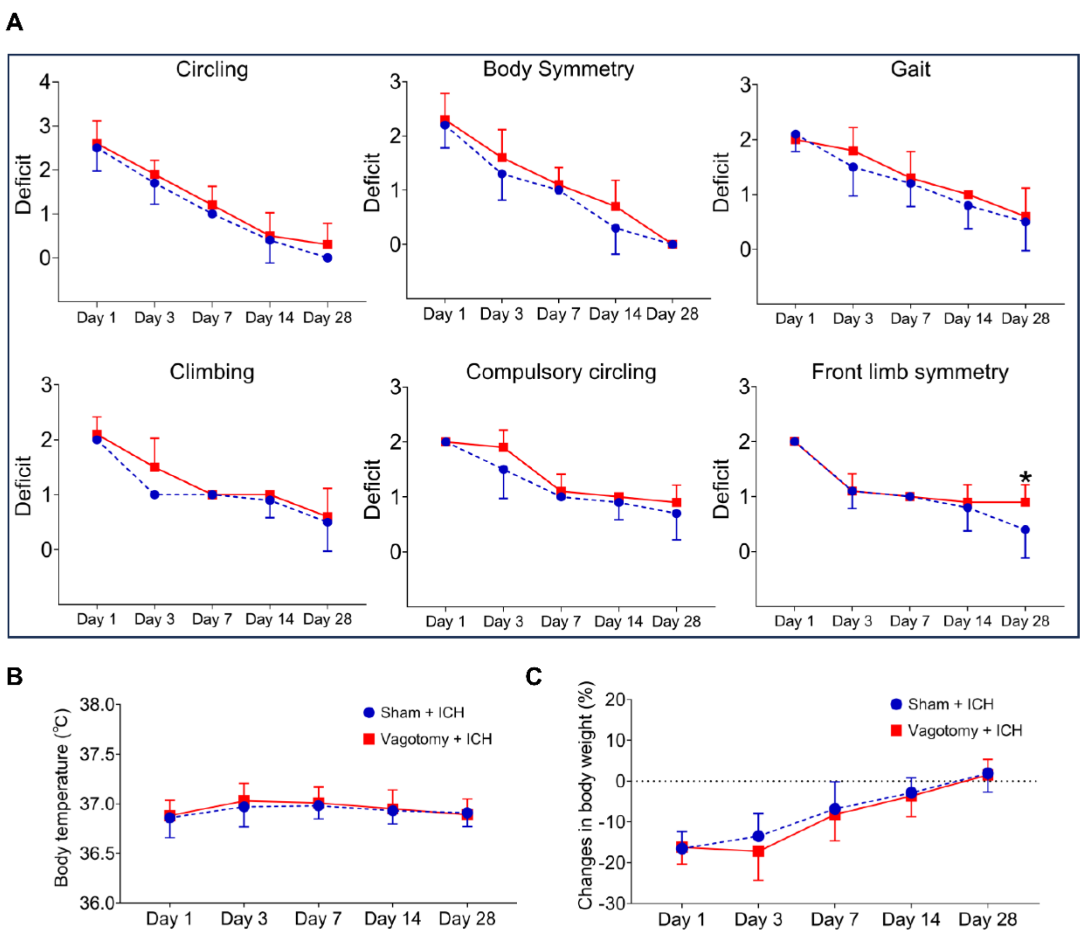

Figure S3. Dynamic changes in individual NDS tests, body weight, and rectal temperature of ICH mice previously underwent SDV or sham vagotomy.

A) During a 28-day research period, individual NDS tests were performed on ICH mice that had undergone a sham vagotomy or SDV. *n* = 10 mice per group. Generalized estimation equations were used, followed by the two-tailed Mann-Whitney U test. However, no significant differences were found. **B)** Similarly, changes in body weight and rectal body temperature were monitored in ICH mice throughout the research period, and we performed the analysis with repeated measures ANOVA of the results. However, no significant differences were found between mice that had undergone a sham vagotomy or SDV. *n* =

10 mice per group.

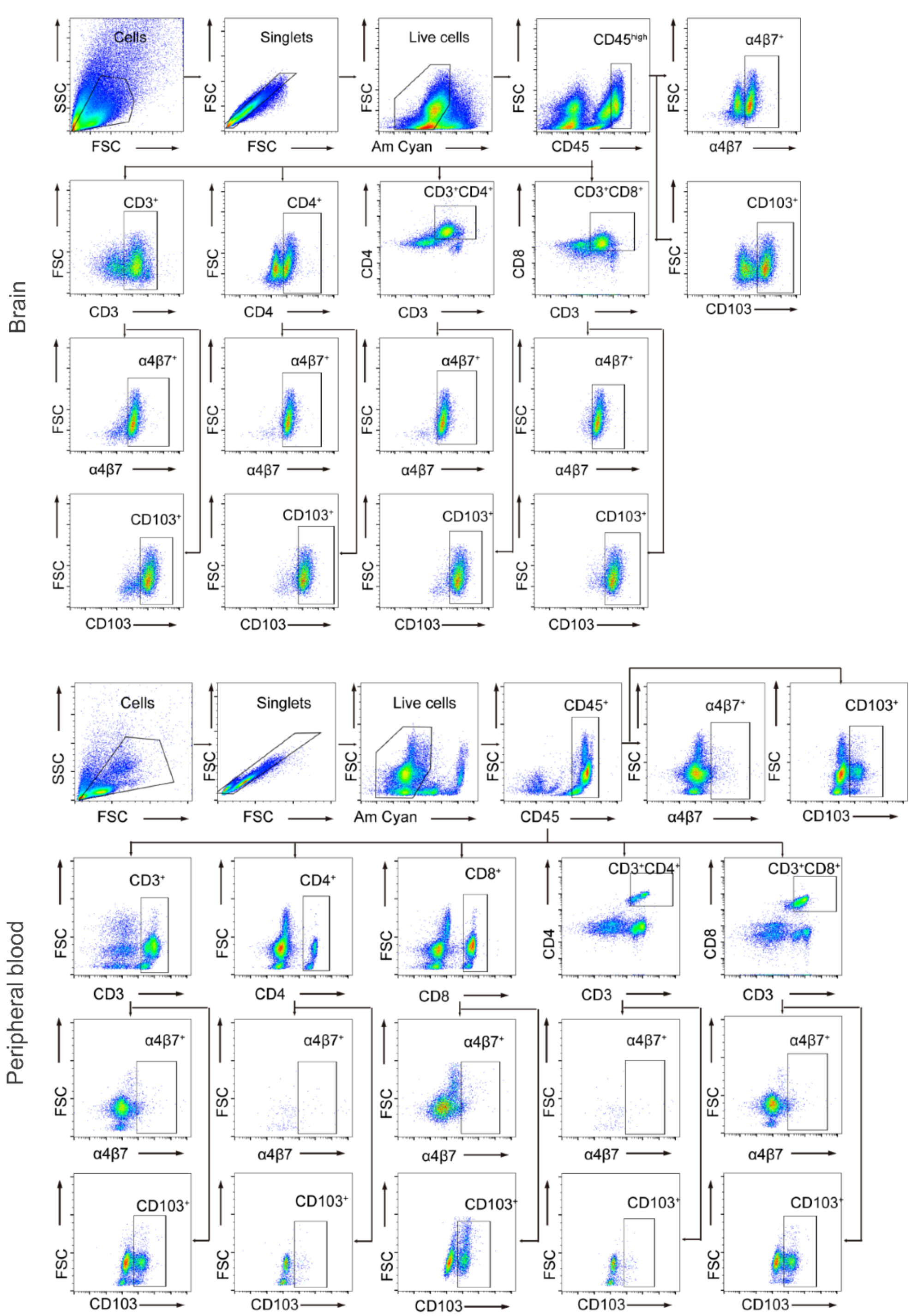

Figure S4. Representative gating strategies for cells isolated from the brain and peripheral blood concerning the effects of α7nAChR agonists on previous SDV in ICH.

A) Representative gating strategies used to detect α4β7 integrin and CD103 in

the populations of $CD45^{high}$, $CD3^+$, $CD4^+$, $CD3^+CD4^+$, and $CD3^+CD8^+$ cells isolated from the brain 3 d after ICH. **B)** Representative gating strategies used to detect α4β7 integrin and CD103 in the populations of $CD45^+$, $CD3^+$, $CD4^+$, $CD8^+$, $CD3^+CD4^+$, and $CD3^+CD8^+$ cells in peripheral blood 3 d after ICH.

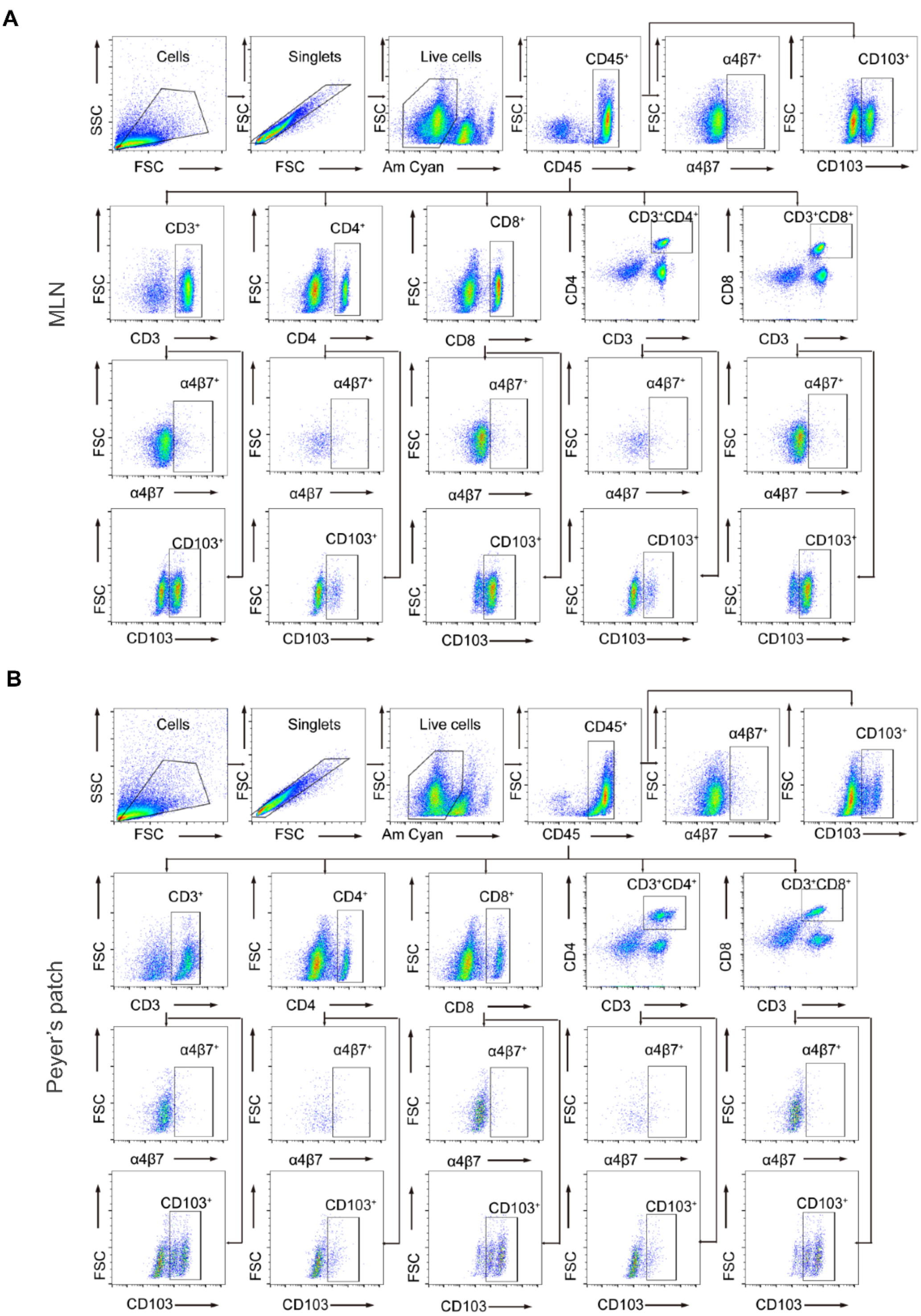

Figure S5. Representative gating strategies of cells isolated from MLNs and Peyer's patches concerning the effects of α7nAChR agonists on previous SDV in ICH.

A) Representative gating strategies used to detect α4β7 integrin and CD103 in

the populations of $CD45^+$, $CD3^+$, $CD4^+$, $CD8^+$, $CD3^+CD4^+$, and $CD3^+CD8^+$ cells isolated from the MLNs 3 d after ICH. **B)** Representative gating strategies used to detect α4β7 integrin and CD103 in the populations of $CD45^+$, $CD3^+$, $CD4^+$, $CD8^+$, $CD3^+CD4^+$, and $CD3^+CD8^+$ cells isolated from Peyer’s patches 3 d after ICH.

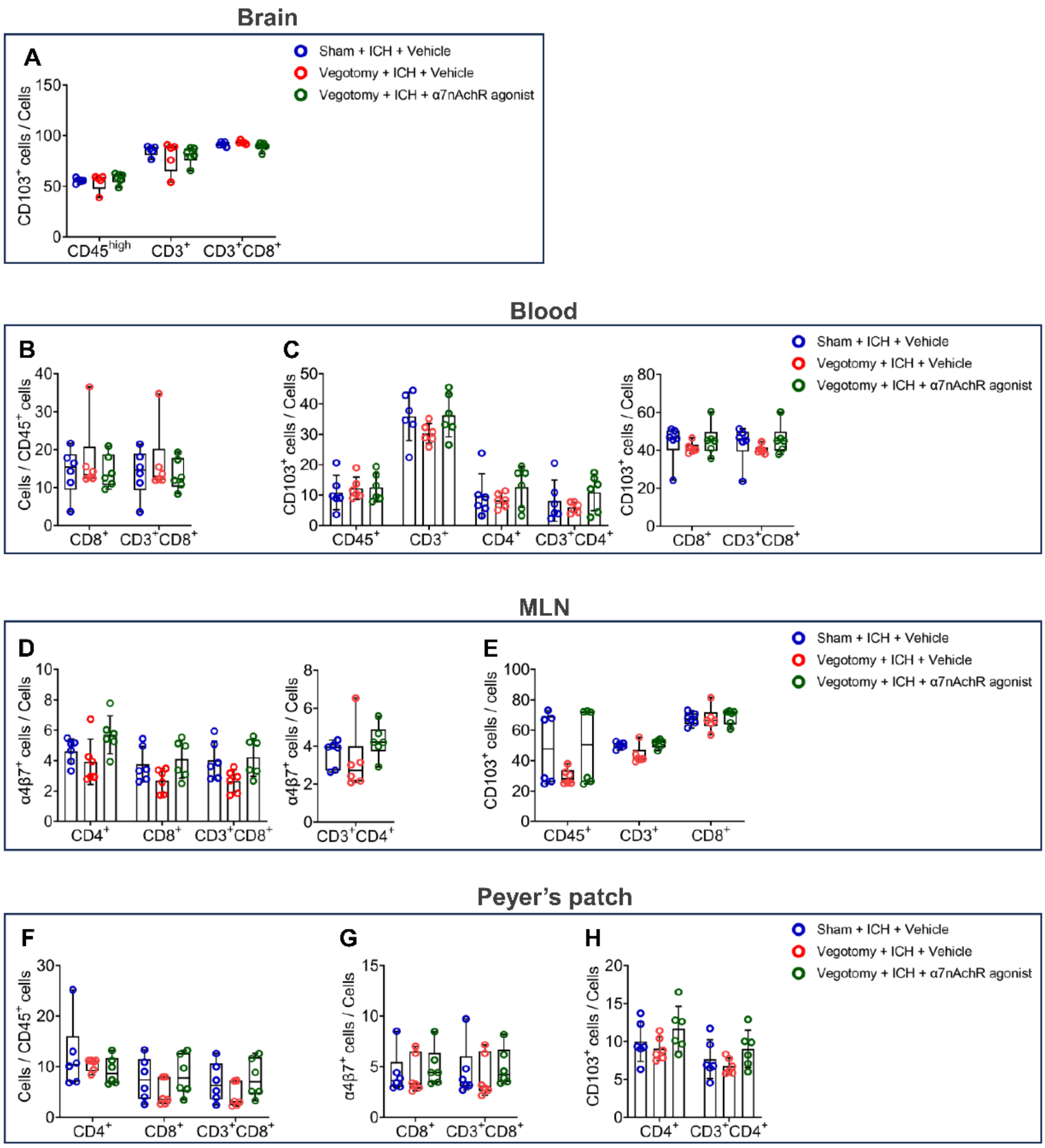

Figure S6. Negative findings on the reversible effects of α7nAChR agonists on intestinal immunocyte homing and retention detected by flow cytometry after SDV in the acute phase of ICH.

A) The influence of previous SDV or previous SDV supplied with α7nAChR agonist treatment on the proportions of CD103-positive $CD45^{high}$, $CD3^{+}$, and $CD3CD8^{+}$ cells in the hemorrhagic brain 3 d after ICH. *n* = 6 mice per group. The Kruskal-Wallis test was performed. **B)** The ratios of $CD8^{+}$ and $CD3^{+}CD8^{+}$ cells to $CD45^{+}$ cells in the peripheral blood 3 d after ICH. *n* = 6 mice per group.

The Kruskal-Wallis test was performed. **C)** Percentage changes of CD103-positive $CD45^+$, $CD3^+$, $CD4^+$, $CD8^+$, $CD3^+CD4^+$, and $CD3^+CD8^+$ cells in the peripheral blood 3 d after ICH. n = 6 mice per group. One-way ANOVA with Bonferroni correction for CD103-positive $CD45^+$, $CD3^+$, $CD4^+$, and $CD3^+CD4^+$ cells, while the Kruskal-Wallis test for others was performed. **D)** Proportional changes of α4β7 integrin-positive $CD4^+$, $CD8^+$, $CD3^+CD4^+$, and $CD3^+CD8^+$ cells in the peripheral blood 3 d after ICH. n = 6 mice per group. The Kruskal-Wallis test for α4β7 integrin-positive $CD3^+CD4^+$, while one-way ANOVA with Bonferroni correction for α4β7 integrin-positive $CD45^+$, $CD8^+$, and $CD3^+CD8^+$ cells was performed. **E)** The proportions of CD103-positive $CD45^+$, $CD3^+$, and $CD8^+$ cells in the MLNs 3 d after ICH. n=6 mice per group. The Kruskal-Wallis test was performed. **F)** The percentages of $CD4^+$, $CD8^+$, and $CD3^+CD8^+$ cells to $CD45^+$ cells in the Peyer's patches 3 d after ICH. The Kruskal-Wallis test was performed. **G)** The ratios of α4β7 integrin-positive $CD8^+$ and $CD3^+CD8^+$ cells in Peyer's patches 3 d after ICH. n = 6 mice per group. The Kruskal-Wallis test was performed. **H)** The percentages of CD103-positive $CD4^+$ and $CD3^+CD4^+$ cells in Peyer's patches 3 d after ICH. n = 6 mice per group. One-way ANOVA with Bonferroni correction was performed. The percentages of the cell subpopulations indicated in this figure were not influenced by previous SDV or previous SDV combined with α7nAChR agonists.

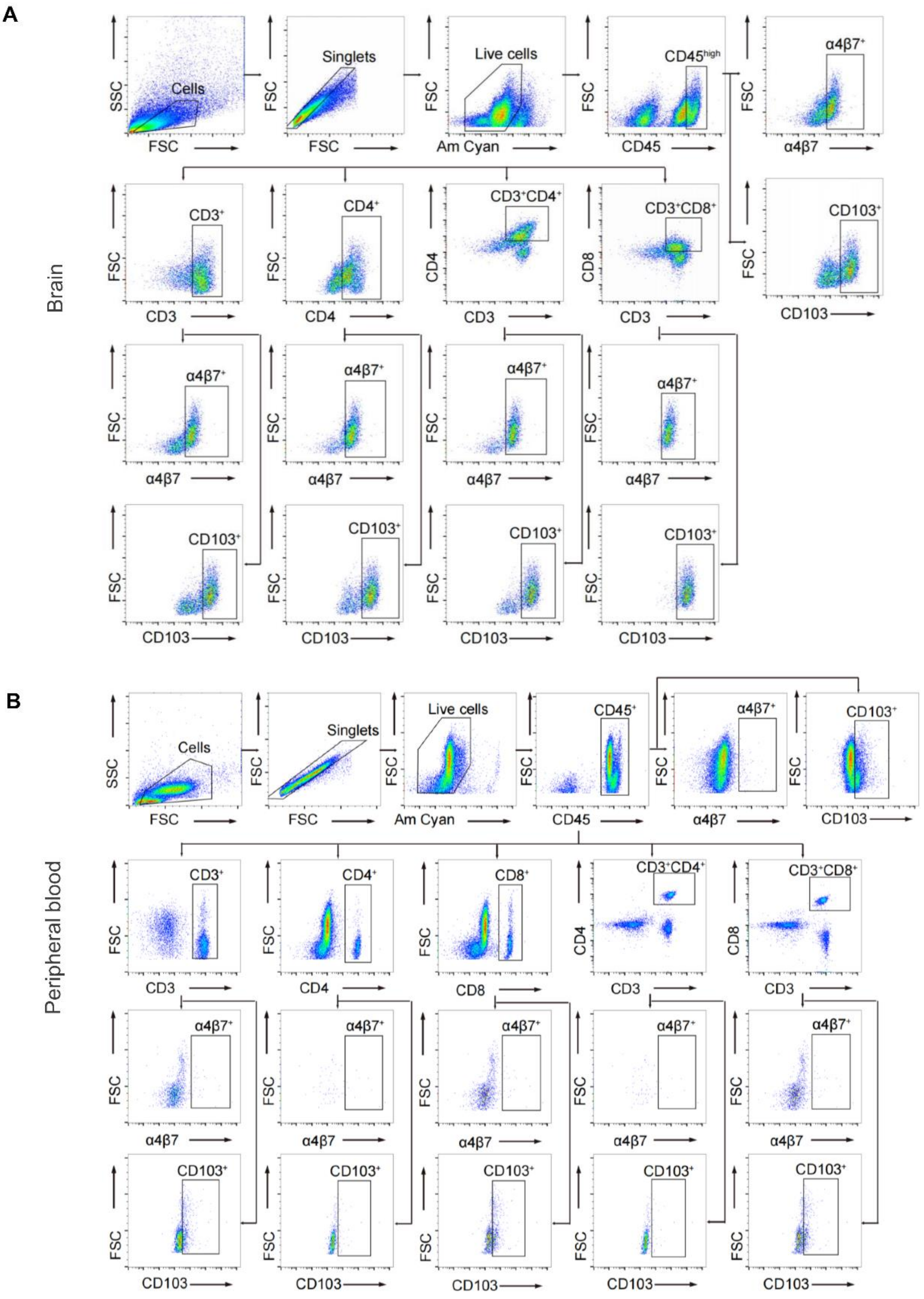

Figure S7. Representative gating strategies for cells isolated from the brain and peripheral blood concerning the effects of α7nAChR agonists on β7 integrin antagonists in ICH.

A) A representative flow cytometric gating strategy for α4β7 integrin and CD103

in the populations of $CD45^{high}$, $CD3^+$, $CD4^+$, $CD3^+CD4^+$, and $CD3^+CD8^+$ cells accumulated to the hemorrhagic brain 3 d after ICH. **B)** The flow cytometry correlation strategies used for α4β7 integrin and CD103 in the populations of $CD45^+$, $CD3^+$, $CD4^+$, $CD8^+$, $CD3^+CD4^+$, and $CD3^+CD8^+$ cells in peripheral blood 3 d after ICH.

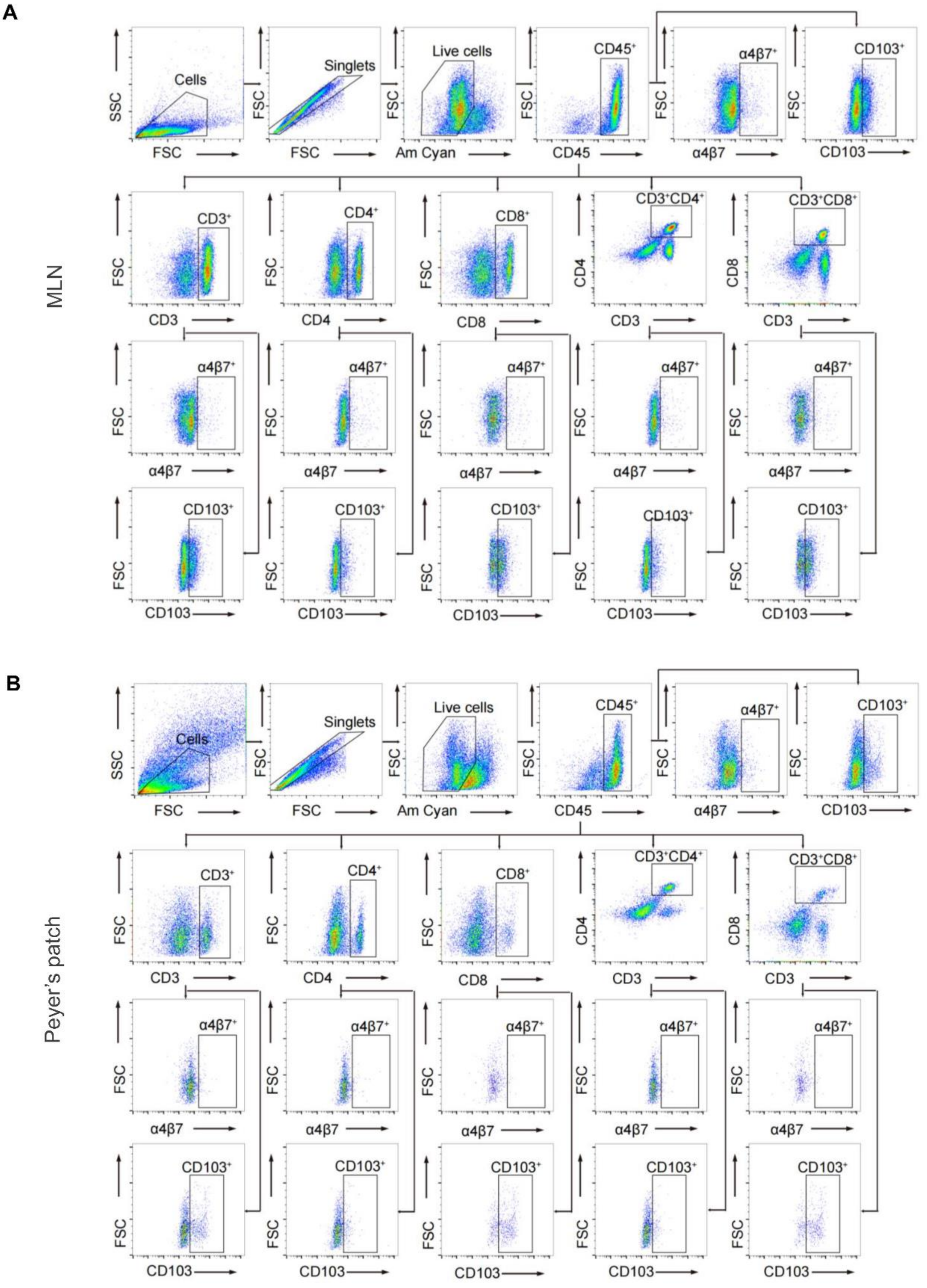

Figure S8. Representative gating strategies of cells isolated from MLNs and Peyer's patches concerning the effects of α7nAChR agonists on β7 integrin antagonists in ICH.

A) The gating strategies for α4β7 integrin and CD103 in the populations of $CD45^+$, $CD3^+$, $CD4^+$, $CD8^+$, $CD3^+CD4^+$, and $CD3^+CD8^+$ cells isolated from the MLNs 3 d after ICH. **B)** Representative flow cytometric gating strategies for α4β7 integrin and CD103 in the populations of $CD45^+$, $CD3^+$, $CD4^+$, $CD8^+$, $CD3^+CD4^+$, and $CD3^+CD8^+$ cells isolated from Peyer's patches 3 d after ICH.

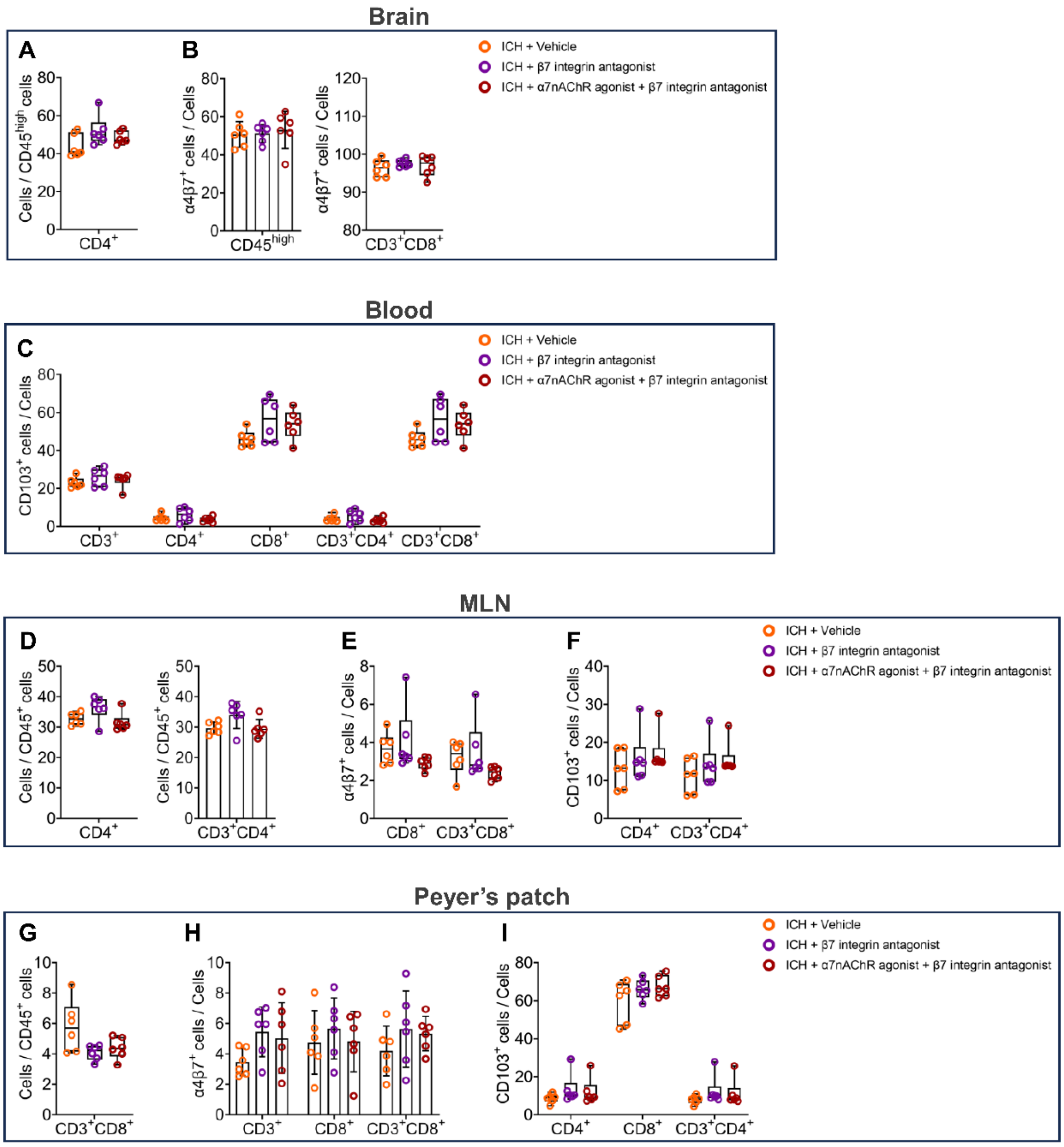

Figure S9. Negative findings on the effects of β7 integrin antagonists on intestinal immunocyte homing and retention detected by flow cytometry in ICH.

A) Analysis for the influence of β7 integrin antagonists or β7 integrin antagonists combined with α7nAChR agonists on the infiltration of $CD4^+$ cells in the brain lesions 3 d after ICH. n = 6 mice per group. The Kruskal-Wallis test was performed. **B)** Proportional changes of α4β7 integrin-positive $CD45^{high}$ and $CD3^+CD8^+$ cells in the hemorrhagic brain 3 d after ICH. n = 6 mice per group.

One-way ANOVA with Bonferroni correction for $CD45^{high}$ cells and the Kruskal-Wallis test for $CD3^+CD8^+$ cells were performed. **C)** The percentages of CD103-positive $CD3^+$, $CD4^+$, $CD8^+$, $CD3^+CD4^+$, and $CD3^+CD8^+$ cells in the bloodstream 3 d after ICH. *n* = 6 mice per group. The Kruskal-Wallis test was performed. **D)** The proportions of $CD4^+$ and $CD3^+CD4^+$ cells to $CD45^+$ cells in the MLNs 3 d after ICH. *n* = 6 mice per group. One-way ANOVA with Bonferroni correction for $CD4^+$ cells and the Kruskal-Wallis test for $CD3^+CD4^+$ cells were performed. **E)** Percentage changes of α4β7 integrin-positive $CD8^+$ and $CD3^+CD8^+$ cells in the MLNs 3 d after ICH. *n* = 6 mice per group. The Kruskal-Wallis test was performed. **F)** Proportional changes of CD103-positive $CD4^+$ and $CD3^+CD4^+$ cells in the MLNs 3 d after ICH. *n* = 6 mice per group. The Kruskal-Wallis test was performed. **G)** The percentage of $CD3^+CD8^+$ cells to $CD45^+$ cells in the Peyer's patches 3 d after ICH. *n* = 6 mice per group. The Kruskal-Wallis test was performed. **H)** Ratios of α4β7 integrin-positive $CD3^+$, $CD8^+$, and $CD3^+CD8^+$ cells in the Peyer's patches 3 d after ICH. *n* = 6 mice per group. One-way ANOVA with Bonferroni correction for multiple comparisons was performed. **I)** The percentages of CD103-positive $CD4^+$, $CD8^+$, and $CD3^+CD8^+$ cells in the Peyer's patches 3 d post-ICH. *n* = 6 mice per group. The Kruskal-Wallis test was performed. Neither β7 integrin antagonists nor β7 integrin antagonists combined with α7nAChR agonists have influence on the cell subpopulations mentioned above.

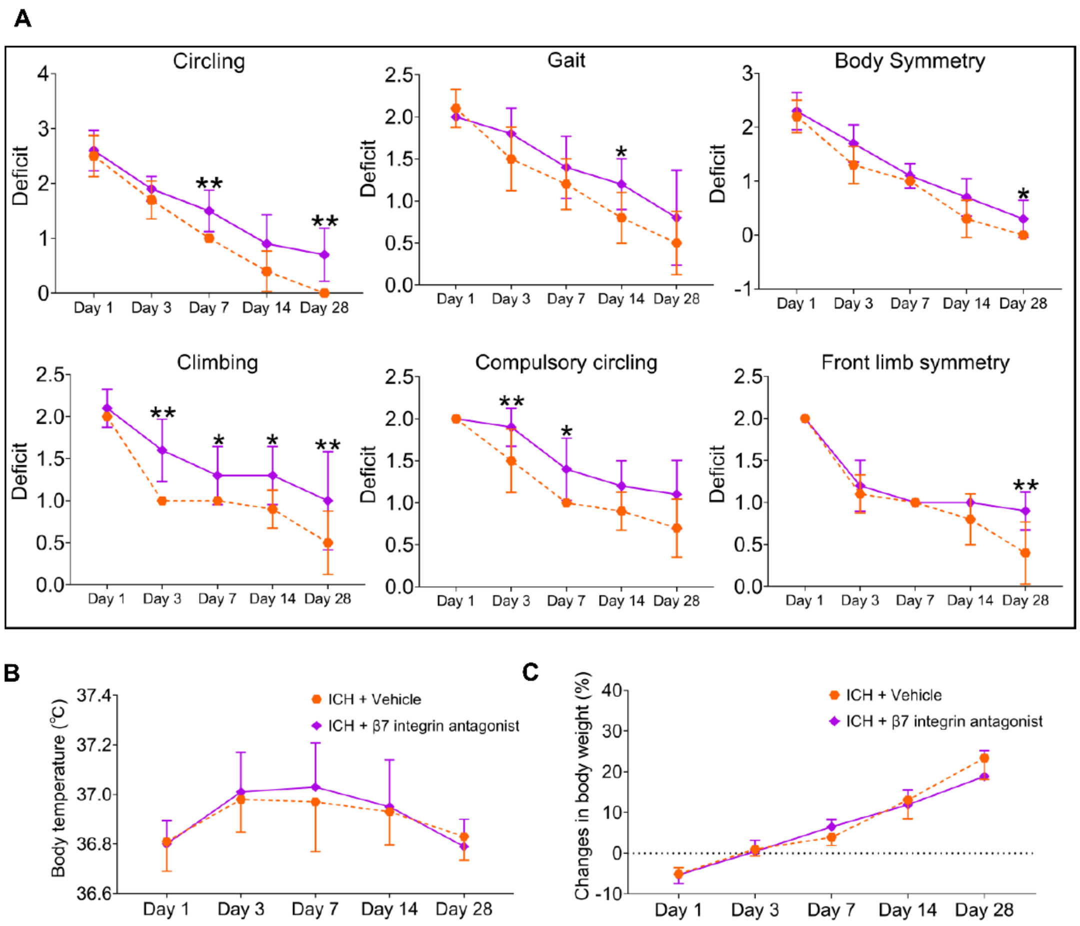

Figure S10. Dynamic changes in individual NDS tests, body weight, and rectal temperature of ICH mice received vehicle or β7 integrin antagonists. **A)** Individual NDS tests on days 1, 3, 7, 14, and 28 after ICH. *n*=10 mice per group. Generalized estimation equations were performed, followed by two-tailed Mann-Whitney U tests. *$P < 0.05$, **$P < 0.01$. **B-C)** Changes in body weight and rectal temperatures at multiple time points during the 28-d research period. *n*=10 mice per group. Generalized estimation equations were performed, followed by two-tailed Mann-Whitney U tests. The results did not show significant differences.
